# Prenatal exposures to organophosphate ester metabolite mixtures and children’s neurobehavioral outcomes in the MADRES pregnancy cohort

**DOI:** 10.1186/s12940-023-01017-3

**Published:** 2023-09-22

**Authors:** Ixel Hernandez-Castro, Sandrah P. Eckel, Caitlin G. Howe, Zhongzheng Niu, Kurunthachalam Kannan, Morgan Robinson, Helen B. Foley, Tingyu Yang, Mario J. Vigil, Xinci Chen, Brendan Grubbs, Deborah Lerner, Nathana Lurvey, Laila Al-Marayati, Rima Habre, Genevieve F. Dunton, Shohreh F. Farzan, Max T. Aung, Carrie V. Breton, Theresa M. Bastain

**Affiliations:** 1 Department of Population and Public Health Sciences, Keck School of Medicine, University of Southern California, 1845 N. Soto Street, Los Angeles, CA USA; 2 Department of Epidemiology, Geisel School of Medicine at Dartmouth, Hanover, NH USA; 3 Wadsworth Center, New York State Department of Health, Albany, NY USA; 4 Department of Obstetrics and Gynecology, Keck School of Medicine, University of Southern California, Los Angeles, CA USA; 5 Eisner Health, Los Angeles, CA USA; 6 Department of Psychology, University of Southern California, Los Angeles, CA USA

**Keywords:** Mixtures, OPE, Organophosphate esters, OPFRs, Neurobehavior, Early childhood

## Abstract

**Background:**

Evidence suggests organophosphate esters (OPEs) are neurotoxic; however, the epidemiological literature remains scarce. We investigated whether prenatal exposures to OPEs were associated with child neurobehavior in the MADRES cohort.

**Methods:**

We measured nine OPE metabolites in 204 maternal urine samples (gestational age at collection: 31.4 ± 1.8 weeks). Neurobehavior problems were assessed among 36-month-old children using the Child Behavior Checklist’s (CBCL) three composite scales [internalizing, externalizing, and total problems]. We examined associations between tertiles of prenatal OPE metabolites (> 50% detection) and detect/non-detect categories (< 50% detection) and CBCL composite scales using linear regression and generalized additive models. We also examined mixtures for widely detected OPEs (*n* = 5) using Bayesian kernel machine regression.

**Results:**

Maternal participants with detectable versus non-detectable levels of bis(2-methylphenyl) phosphate (BMPP) had children with 42% (95% CI: 4%, 96%) higher externalizing, 45% (-2%, 114%) higher internalizing, and 35% (3%, 78%) higher total problems. Participants in the second versus first tertile of bis(butoxethyl) phosphate (BBOEP) had children with 43% (-1%, 109%) higher externalizing scores. Bis(1-chloro-2-propyl) phosphate (BCIPP) and child sex had a statistically significant interaction in internalizing (*p* = 0.02) and total problems (*p* = 0.03) models, with 120% (23%, 295%) and 57% (6%, 134%) higher scores in the third versus first BCIPP tertile among males. Among females, detectable vs non-detectable levels of prenatal BMPP were associated with 69% higher externalizing scores (5%, 170%) while the third versus first tertile of prenatal BBOEP was associated with 45% lower total problems (-68%, -6%). Although the metabolite mixture and each CBCL outcome had null associations, we observed marginal associations between di-n-butyl phosphate and di-isobutyl phosphate (DNBP + DIBP) and higher internalizing scores (0.15; 95% CrI: -0.02, 0.32), holding other metabolites at their median.

**Conclusions:**

Our results generally suggest adverse and sex-specific effects of prenatal exposure to previously understudied OPEs on neurobehavioral outcomes in 36-month children, providing evidence of potential OPE neurotoxicity.

**Supplementary Information:**

The online version contains supplementary material available at 10.1186/s12940-023-01017-3.

## Background

Neurobehavioral development is a lifelong, dynamic process which encompasses a host of psychosocial and biological processes that influence behavior, emotion, and learning [1, 2]. Environmental chemical exposures are increasingly recognized as major risk factors for adverse neurobehavioral outcomes, ranging in effects from subclinical deficits in neurobehavioral functioning to increased risks of neurobehavioral disorders [2–4]. The prenatal period is a particularly susceptible window for neurobehavioral development given the rapid cascade of tightly controlled and sequenced biological processes that occur in utero, resulting in heightened susceptibility to environmental exposures [2]. Even minor, incremental disruptions to prenatal biological processes from low-level chronic exposures to environmental chemicals have the potential to result in lifelong health effects [3, 5].

Flame retardants are anthropogenic chemical additives incorporated into materials to prevent or delay fires and to meet flammability regulations in the United States, particularly in California [6, 7]. For many decades, legacy flame retardants, such as polybrominated diphenyl ethers (PBDEs), were the most frequently used [8, 9]. However, due to their bioaccumulation in the environment, persistence, and neurotoxicity to children, PBDEs have been phased out of the US market and banned from production in the European Union [10]. As a result, organophosphate esters (OPEs) have dramatically increased in use as replacement flame retardants in recent years [11–13]. However, emerging literature suggests that OPEs may be a regrettable substitution for PBDEs and may also adversely impact neurobehavioral and neurodevelopmental outcomes [14].

OPEs are commonly used as plasticizers and lubricants, contributing to their environmental ubiquity [7]. OPEs are also applied as additives to various consumer, industrial, and electronic products, such as polyurethane foam, textiles, and building materials [7, 15]. Due to their physical incorporation within a product matrix and their semivolatile nature, OPEs easily volatize and leach into surrounding environments, commonly settling into dust particles in homes and environmental media such as soil, surface water, sediment, and agricultural products and facilitating human exposure to OPEs [16–24]. As a result, common OPE exposure routes include dermal contact, inhalation, and ingestion of air and dust particles, as well as dietary ingestion of OPE-contaminated food and drinking water [7, 15]. OPEs have been found in the placenta and cord blood, suggesting in utero transfer to the fetus, and resulting in growing concern, particularly regarding early neurodevelopment, given the structural similarity between OPEs and organophosphate pesticides which have been previously found to be neurotoxic [25–30]. The two most frequently detected OPE metabolites among people in the U.S are diphenyl phosphate (DPHP; parent compound, triphenyl phosphate (TPHP)) and bis(1,3-dichloro-2-propyl) phosphate (BDCIPP; parent compound, tris(1,3-dichloropropyl) (TDCIPP)), with greater than 95% detection frequencies in the 2013 to 2014 National Health and Nutrition Examination Survey [31, 32].

Growing experimental and observational evidence suggests that OPEs may affect early behavioral development at environmentally relevant doses via multiple biological mechanisms, including inflammation of various neuropathways, neurotransmitter perturbations, oxidative stress, and endocrine disruption [33–37]. Limited studies have reported associations between prenatal OPE exposures and increased externalizing behaviors, such as rule-breaking and aggression, and attention problems in children, with most of those studies only examining the effects of the two most common OPE metabolites, BDCIPP and DPHP [38, 39]. Similarly, studies have primarily examined the impacts of these single OPEs on neurobehavioral symptoms, rather than co-occurring impacts of multiple OPE exposures which are more representative of daily exposures mixtures [38, 40]. A more thorough understanding of the impacts of prenatal OPE exposures on early neurobehavior is critical to developing appropriate interventions and regulations to mitigate neurotoxic exposures.

This study’s objective was to evaluate the impacts of nine urinary prenatal OPE metabolites individually and as a mixture on a broad range of early childhood neurobehavioral symptoms, including internalizing, externalizing, and total problems, among mother-infant dyads participating in the MADRES cohort study. We hypothesized that higher prenatal exposures to OPE metabolites and OPE metabolite mixtures adversely impact child neurobehavioral outcomes at 36 months of age.

## Methods

### Study design

The MADRES study is an ongoing prospective pregnancy cohort of predominately low-income Hispanic/Latino mother-child pairs living in urban Los Angeles, CA. A detailed description of the MADRES study population and protocol have been previously described [41]. In brief, participants were recruited into the study prior to 30 weeks’ gestation at three partner community health clinics, one private obstetrics and gynecology practice in Los Angeles, and through self-referrals from community meetings and local advertisements. Eligible participants at time of recruitment were: (1) less than 30 weeks’ gestation, (2) over 18 years of age, and (3) fluent in English or Spanish. Exclusion criteria included: (1) multiple gestation, (2) having a physical, mental, or cognitive disability that prevented participation or ability to provide consent, (3) current incarceration, and (4) HIV positive status. Written informed consent was obtained at study entry for each participant and the study was approved by the University of Southern California’s Institutional Review Board.

**Fig. 1:**
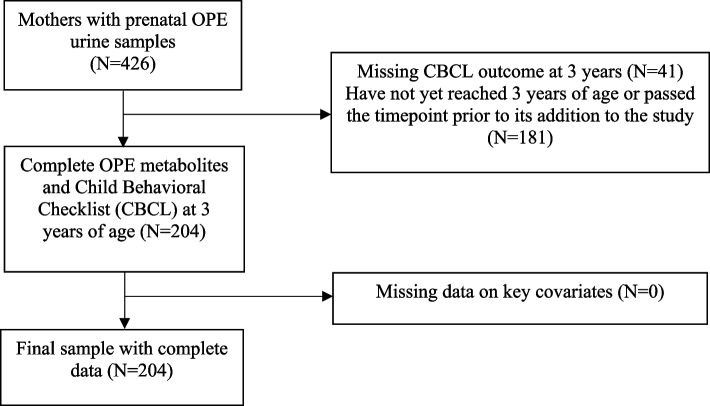
Consort diagram of included mother-infant dyads

Nine urinary OPE metabolite concentrations were measured in 426 participants’ urine samples provided during the third trimester study visit (mean GA at sample collection ± SD = 31.4 ± 1.8 weeks) from 2017 to 2019. Child neurobehavior was assessed using the Child Behavioral Checklist 1.5–5 years (CBCL 1.5–5) composite scales, including the internalizing problems, externalizing problems, and total problems scales, administered at the 36-month timepoint. As shown in the consort diagram (Fig. 1), mother-child participants with complete information on the exposure, outcome, and key covariates of interest were included in the final analytic sample. A total of 204 mother-child dyads with available data on OPE metabolite concentrations, the CBCL administered at 36 months, and key covariates were included in this study.

### OPE metabolites

Single spot urine samples were collected in 90 mL sterile specimen containers during a third trimester study visit. Urine specimens were aliquoted into 1.5 mL aliquot cryovials and specific gravity was measured in room temperature urine samples using a digital handheld refractometer (ATAGO PAL-10s pocket refractometer). Samples were stored at -80 ºC prior to shipment and sent to the Wadsworth Center’s Human Health Exposure Analysis Resource (HHEAR) lab hub for the analysis of the following nine OPE metabolites: diphenyl phosphate (DPHP), composite of di-n-butyl phosphate and di-isobutyl phosphate (DNBP + DIBP), bis(1,3,-dichloro-2-propyl) phosphate (BDCIPP), bis(2-chloroethyl) phosphate (BCEP), bis(butoxethyl) phosphate (BBOEP), bis(1-chloro-2-propyl) phosphate (BCIPP), bis(2-ethylhexyl) phosphate (BEHP), bis(2-methylphenyl) phosphate (BMPP), and dipropyl phosphate (DPRP). Additional information on each metabolite, the corresponding parent compound, and common uses are described in Table S1.

Urinary OPE metabolites were quantified following methods similar to those previously described, with some slight modifications [42]. In brief, urine samples (0.5 mL) were aliquoted into pre-baked glass tubes and spiked with 1 ng of deuterated internal standard (IS) mixtures of OPEs and 1 mL of 10 mM ammonium acetate buffer (pH 5). The samples were passed through solid phase extraction (SPE) cartridges (STRATA-X-AW: 60 mg, 3 cc, Phenomenex, Torrance, CA, USA) which were conditioned by successive passage with 2 mL of 5% (v/v) ammonia/methanol, 2 mL of methanol, and 2 mL of water. The samples were loaded with the valves partially opened. The SPE cartridges were then dried under vacuum for 3 min after washing with 1.0 mL of water. Analytes were eluted with 2 times 0.5 mL of 5% (v/v) ammonia/methanol, concentrated under a gentle stream of nitrogen at 37 °C to near dryness, and reconstituted with 0.1 mL of acetonitrile.

High-performance liquid chromatography (HPLC, ExionLC™ system; SCIEX, Redwood City, CA, USA), coupled with an AB SCIEX QTRAP 5500+ triple quadrupole mass spectrometer (TQMS, Applied Biosystems, Foster City, CA, USA), was used in the identification and quantification of target compounds. Nine OPE diester metabolites and corresponding 9 internal standards were separated by a Kinetex HILIC column (100 mm × 2.1 mm, 2.6 μm particle size; Phenomenex) serially connected to a Betasil C18 guard column (20 mm × 2.1 mm, 5 μm particle size; Thermo Scientific). The analytes were quantified by isotopic dilution method and an 11-point calibration curve (at concentrations ranging from 0.02 to 50 ng/mL) with the regression coefficient ≥ 0.998. Matrix spikes (synthetic and urine pool spiked with 1 ng of native standards and 1 ng of internal standards) were analyzed with real samples as quality control (QC) samples. For each batch of samples, replicates of reagent blanks, matrix blanks, and matrix spiked samples were processed. Replicates of HHEAR Urine Quality Control (QC) Pools Standard Reference Materials (SRM3672 and SRM3673, NIST, Gaithersburg, MD, USA) were analyzed with every batch of samples. Trace levels of all OPE diester metabolites were found in procedural blanks. OPE diester metabolite concentrations measured in blanks were subtracted from sample values. Matrix spiked samples had average recoveries of 70.4–133% (CV: ± 9–19%). Repeated analysis of HHEAR Urine QC Pools A and B among batches showed coefficients of variation of ± 12–31% and ± 12–30% respectively. SRM3672 and SRM3673 had coefficients of variation of ± 12–40% and ± 12–27% respectively. Target analytes limit of detection (LOD) ranged from 0.012 to 0.044 ng/mL. Due to poor chromatographic separation and co-elution of peaks accompanying a similar mass transition for DNBP and DIBP, these two isomers were reported as sum concentration of di-n-butyl phosphate and di-isobutyl phosphate (DNBP + DIBP).

OPE metabolites with concentrations below the LOD were imputed using the LOD/$\surd 2$ [43]. Metabolites were then specific gravity (SG) adjusted using the following formula: Pc = P[(SGm-1)/(SG-1)], where Pc is the specific gravity corrected toxicant concentration (ng/mL), P is the observed toxicant concentration (ng/mL), SGm is the median SG value among the study population (median = 1.016), and SG = the SG value of the sample.

### Health outcome assessment

The Child Behavior Checklist for ages 1½ through 5 years old (CBCL 1.5–5) is a 99-item questionnaire which has been validated and widely used to assess a broad range of emotional and behavioral problems in children [44]. The questionnaire was orally administered to maternal participants during the 36 month study visit who indicated the frequency of behaviors in their child within the prior 2 months on a 3-point Likert scale (not true = 0, sometimes true = 1, or very often true = 2), with each raw scale created by summing together relevant items and t-scores and corresponding borderline (t-scores: 60–63) and clinical symptom categories (t-scores: ≥ 64) calculated based on previously described criteria to quantify areas that may warrant evaluation by a professional [45]. Higher scores across all CBCL scales indicate increasing problems. The CBCL consists of seven scored syndrome scales (emotionally reactive (9 items), anxious/depressed (8 items), somatic complaints (11 items), withdrawn (8 items), sleep problems (7 items), attention problems (5 items), aggressive behavior (19 items), and other problems (33 items)). These syndrome scales can be summed to create two composite scales, internalizing problems (emotionally reactive, anxious/depressed, somatic complaints, and withdrawn) and externalizing problems (attention problems and aggressive behavior). The CBCL additionally includes a total problems score which is the summed total of all 99 questionnaire items, plus the highest score on any additional problems listed under an open-ended item, question 100 (score range = 0–200). For the purposes of this analysis, the raw internalizing problems, externalizing problems, and total problems scores were each analyzed to encapsulate the breadth of potential behavioral and emotional developmental problems experienced by participants and to facilitate comparisons to prior studies similarly examining impacts of OPEs on raw CBCL scores [40]. However, sensitivity analyses examining associations between OPEs and CBCL t-scores were also evaluated to assess the robustness of our results after standardizing raw scores to a normative US sample of children.

### Covariates

Covariates assessed in this analysis were study design or sample collection variables or were identified based on previous literature which examined impacts of neurotoxic chemicals on early neurobehavioral development [3, 31, 39, 40]. Relationships between prenatal OPE metabolites and neurobehavioral development were visualized using a Directed Acyclic Graph (DAG) created using DAGitty (Fig. S1) [46]. All models were adjusted for variables identified in the DAG’s minimal sufficient adjustment set (maternal age, parity, pre-pregnancy BMI, race/ethnicity, income, and education) and study design or sample collection variables whose inclusion in models changed the effect estimate of our exposure of interest by 10% or more (recruitment site, specimen collection season, GA at sample collection, and child adjusted age at CBCL administration). The only exception to these criteria was adjustment for maternal-reported smoking during pregnancy. Prenatal smoking was identified in the minimal sufficient adjustment set, but, given the small frequency of maternal smoking (*n* = 5, 2.5%), we instead evaluated its impact in sensitivity analyses by removing participants who reported smoking during pregnancy. Additionally, child sex was adjusted for in all models since it is an important predictor of neurobehavioral outcomes and was also evaluated as an effect modifier in adjusted models.

Maternal age (years), household annual income during pregnancy (< $50,000, ≥ $50,000, do not know), education (≤ 12^th^ grade, > 12^th^ grade), race/ethnicity (White non-Hispanic, Black non-Hispanic, Hispanic, Multiracial non-Hispanic/Other non-Hispanic), maternal smoking during pregnancy (yes, no), and parity (first born, ≥ second born, missing) were collected via interviewer administered questionnaires in the participant’s preferred language (English or Spanish). Pre-pregnancy BMI was calculated using participant-reported pre-pregnancy weight and standing height measured by study staff at the first study visit using a commercial stadiometer (Perspectives Enterprises model P-AIM-101). Child sex assigned at birth was primarily abstracted from electronic medical records (*n* = 200, 98.0%), followed by maternal-reported child sex (*n* = 4, 2.0%) for cases in which abstracted sex could not be obtained. Child adjusted age at time of questionnaire administration was calculated in weeks using date of birth and date of questionnaire administration, corrected for premature birth (< 37 weeks).

### Statistical analysis

We examined participant demographic characteristics using means and frequencies. OPE metabolite distributions were explored using histograms, geometric means, percentile distributions, and metabolite detect frequencies. Given the generally right skewed distribution of OPE metabolites, Kruskal Wallis tests were conducted to evaluate bivariate associations between categorical covariates and OPE concentrations and Spearman correlations were performed to evaluate associations between OPE metabolites.

The distribution of CBCL raw scores was right skewed with 7.4% and 2.5% of scores with a 0 on the internalizing and externalizing problems scales, respectively; therefore, CBCL scores were offset by 0.1 and natural log transformed prior to linear regression modeling. Locally Weighted Scatterplot Smoothing (LOWESS) plots between prenatal OPEs and CBCL composite scales were then evaluated, and due to non-linear associations that persisted after natural log transformation, OPE metabolites were categorized into exposure tertiles prior to linear regression modeling. For OPE biomarkers detected in > 80% of participants (DPHP, DNBP + DIBP, BDCIPP), OPE metabolites were categorized into tertiles of specific gravity adjusted exposure concentrations. For OPE metabolites detected in 50–80% of participants (BCEP, BBOEP, BCIPP), a three-level categorical variable was created, with the lowest category defined as concentrations < LOD, and the remaining detected values categorized as < median or ≥ median. For OPE biomarkers detected in < 50% of participants (BMPP, BEHP, DPRP), we modeled OPE biomarkers as binary variables that were detected (> LOD) or not detected (≤ LOD). Modeling assumptions for all linear regressions were evaluated and met. A statistical interaction between each OPE metabolite and child sex was also tested in linear regression models. Data were managed and linear regression models were analyzed using SAS v9.4 (SAS Institute, Inc., Cary, NC, USA).

Generalized Additive Models (GAMs) with a smoothing term for natural log transformed OPE metabolites were also performed to evaluate possible non-linear associations between OPE metabolites and neurobehavioral outcomes using the R package “mgcv”. Consistent with prior literature, only metabolites with a detect frequency > 60% (DPHP, DNBP + DIBP, BDCIPP, BCEP, BBOEP) were evaluated using GAMs [47–49]. A statistical interaction between each OPE metabolite and child sex was also tested within independent GAM models, using a factor smooth interaction, and sex-specific exposure smooths were further evaluated. The significance level for single chemical analysis models was set at an alpha of 0.05.

Bayesian kernel machine regression (BKMR) was selected as the primary mixture modeling approach given its ability to: 1) accommodate non-linear associations between an exposure and outcome of interest, while accounting for potential correlated exposures, and 2) evaluate possible synergistic and antagonistic relationships between mixtures components without prior specification [50, 51]. Only metabolites with a detect frequency > 60% were included in BKMR models (*n* = 5 metabolites), consistent with prior studies [49]. BKMR is an advanced semi-parametric method which uses Gaussian kernel machine regression to estimate the effects of a high-dimensional matrix of predictors (e.g., interrelated environmental exposures) on a health outcome of interest [50]. The BKMR model for the current study is represented by the following equation:

$${Y}_{i}=h\left({DPHP}_{i},{DNB{P}_{i}+DIBP}_{i},{BDCIPP}_{i}, {BCEP}_{i}, {BBOEP}_{i}\right)+{X}_{i}\beta +{\varepsilon }_{i}$$

where ${Y}_{i}$ represents our health outcome of interest (i.e., internalizing problems, externalizing problems, and total problems) for participant i, $h$(.) denotes the exposure-response function; $\beta$ represents the vector of coefficients for model covariates (${X}_{i}$), which are modeled parametrically; and $\varepsilon$ represents residuals assumed to be independent, normally distributed, with a common variance. Five OPE metabolites detected in > 60% of samples and CBCL raw composite scales were natural log transformed, mean-centered, and standard deviation scaled prior to BKMR modeling to facilitate comparisons. All continuous covariates were mean centered and scaled to one standard deviation.

The overall effect of the OPE mixture on each CBCL composite scale was evaluated by assessing the expected change in each score associated with concurrently increasing percentiles of all metabolites (DPHP, DNBP + DIBP, BDCIPP, BCEP, BBOEP), relative to fixing all metabolites at their median. If the 95% credible interval (CrI) did not span 0, we considered the metabolite or mixture to be associated with the outcome. Posterior inclusion probabilities (PIPs) were also estimated to assess the relative importance of each metabolite in the joint mixture effect with each CBCL composite raw score. Cross sections of the high-dimensional exposure-response functions were plotted for each OPE holding all other exposures constant at their 50^th^ percentiles to assess the shape, direction, and magnitude of association between each OPE metabolite, accounting for the rest of the mixture, with the CBCL composite scales. We also estimated the effect of an increase from the 25^th^ to the 75^th^ percentile of a single metabolite on each CBCL composite scale when all other metabolites were fixed at the median. Possible pairwise interactions between OPE metabolites were also investigated visually for each CBCL composite scale by assessing the association between each OPE metabolite and outcome when varying a second OPE metabolite to its 25^th^, 50^th^, and 75^th^ percentile (holding all other OPE metabolites at their 50^th^ percentile) with non-parallel lines indicating possible pairwise interactions.

The bkmr R package (R v.4.1) was used for the BKMR analysis [51]. The Markov chain Monte Carlo (MCMC) sampler was used to obtain 100,000 posterior samples of model parameters, with the first half of iterations used as burn-in and chains thinned to every 10^th^ iteration to reduce potential autocorrelation. Visual inspection of trace plots and the Gelman-Rubin statistic were used to evaluate convergence, with both trace plots and Gelman-Rubin values below 1.1 indicating convergence. BKMR models were assumed to have non-informative prior distributions in primary models, the default specified in the R package.

In order to further investigate possible synergistic and antagonistic relationships between OPE metabolites, a new Bayesian semiparametric regression was used to generate PIPs for interactions, using the NLinteraction R package [52]. This analysis was conducted by specifying 100,000 MCMC iterations, with half removed for burn-in and default options selected. The natural cubic splines used in this method to model the exposure-response relationship were based on the lowest value of Watanabe-Akaike information criterion (WAIC), with the lowest WAIC for the internalizing and total problems models observed at 1 degree of freedom and the lowest WAIC for the externalizing model observed at 2 degrees of freedom. Pairwise interactions with the highest ranked PIPs were then further examined using GAMs, which allowed for a tensor interaction between the pair of metabolites (both evaluated continuously) and adjusted for the individual smoothed term of each metabolite and other covariates to obtain a *p*-value for the interaction. If interaction *p*-values were statistically significant (*p* < 0.05) using GAMs, these relationships were further explored in models in which individual smoothed terms for one of the metabolites were assessed by tertiles of the second metabolite (and vice versa) to facilitate comparisons with the pairwise patterns observed in the BKMR analysis.

### Sensitivity analysis

Various sensitivity analyses were performed to assess the robustness of our results. Models excluding maternal participants who smoked during pregnancy were performed. An additional sensitivity analysis evaluating the effects of CBCL composite t-scores as an alternative parameterization of the CBCL raw scores was also performed. Since BKMR is sensitive to prior distributions, sensitivity analyses varying the parameter which controls the smoothness of the exposure-response association (b) were conducted; we explored both a lower (b = 50) and higher (b = 1000) degree of smoothness. Similarly, given NLinteraction’s sensitivity to model priors, we evaluated the impacts of varying the threshold parameter from the default (0.10) to a less conservative value (0.25). The threshold parameter influences the likelihood of metabolite inclusion into the function. We also performed a sensitivity analysis to assess the impacts of OPE mixtures with a detect frequency > 80% (DPHP, DNBP + DIBP, BDCIPP) on neurobehavioral outcomes.

## Results

### Descriptive statistics

The maternal and infant characteristics of participants analyzed in this study are shown in Table 1. Maternal participants were an average of 29.4 ± 5.9 years old at study recruitment, had an average pre-pregnancy BMI of 29.1 ± 6.5 kg/m^2^, and were predominately Hispanic (78.9%). More than half of participants completed at most high school (55.4%) and had an annual household income of less than $50,000 during pregnancy (57.8%), and only 2.5% (*n* = 5) of maternal participants reported smoking during pregnancy. Most infants were born full term, with an average gestational age at birth of 39.1 ± 1.5 weeks. The distribution of OPE metabolite concentrations was similar in this analytical sample of participants compared with the full subset of 426 MADRES participants with prenatal OPE metabolites analyzed (Table S2). Similarly, this analytical subset was similar to both MADRES participants in the full cohort with children delivered during the study as of August 28, 2022 (*n* = 774) and subset of MADRES participants with OPEs analyzed (*n* = 426) for key demographic characteristics including income, ethnicity, maternal age, education, child sex, and infant GA at birth (see Table S3).

**Table 1:** Participant characteristics (*N* = 204)

	Mean (SD)/Freq(%)
**Maternal Characteristics**	
Age (years)	29.4 (5.9)
Education	
≤ High School	113 (55.4%)
≥ Technical school, college degree, or graduate studies	91 (44.6%)
Income	
Do not Know	55 (27.0%)
Less than $50,000	118 (57.8%)
≥ $50,000	31 (15.2%)
NIH Race Categories	
White, non-Hispanic	17 (8.3%)
Black, non-Hispanic	21 (10.3%)
Hispanic	161 (78.9%)
Multiracial/other, non-Hispanic	5 (2.5%)
Smoking During Pregnancy	
No	199 (98.0%)
Yes	5 (2.5%)
Pre-pregnancy BMI (kg/m^2^)	29.1 (6.5)
**Infant Characteristics**	
Sex	
Female	105 (51.5%)
Male	99 (48.5%)
Infant Birth Order	
First Born	74 (36.3%)
Second or more	123 (60.3%)
Missing	7 (3.4%)
Gestational Age at Birth (weeks)	39.1 (1.5)
Child Adjusted Age at CBCL Administration (weeks)^a^	155.8 (2.3)

^a^Child age at questionnaire administration corrected for preterm birth (< 37 weeks)

**Fig. 2:**
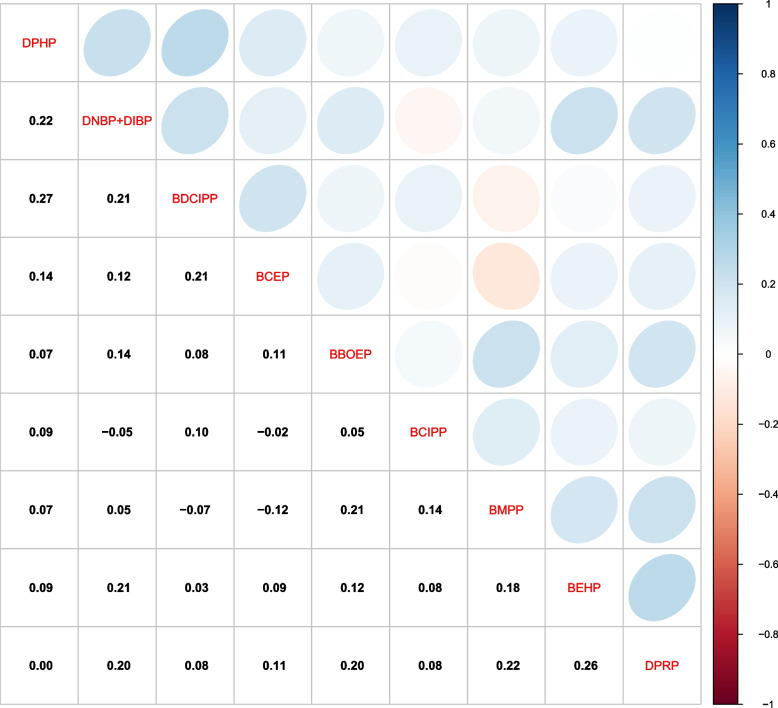
Spearman correlations of organophosphate ester metabolites (ng/mL) in third trimester maternal urine. Note: DPHP, Diphenyl phosphate; DNBP + DIBP, Sum of Di-n-butyl phosphate and Di-isobutyl phosphate; BDCIPP, Bis(1,3-dichloro-2-propyl) phosphate; BCEP, Bis(2-chloroethyl) phosphate; BBOEP, Bis(butoxethyl) phosphate; BCIPP, Bis(1-chloro-2-propyl) phosphate; BMPP, Bis(2-methylphenyl) phosphate; BEHP, Bis(2-ethylhexyl) phosphate; DPRP, Dipropyl phosphate

**Fig. 3:**
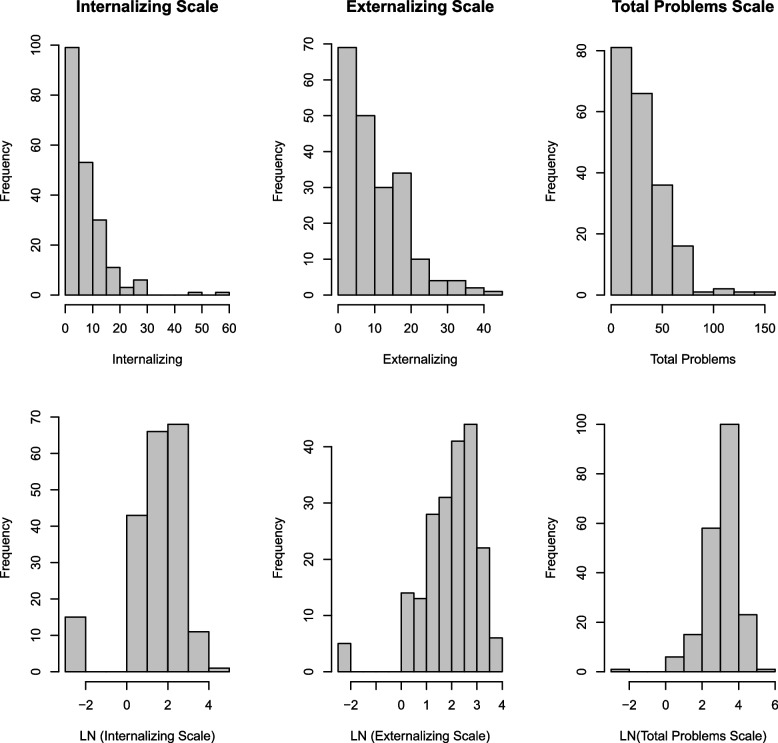
Distributions of 36 month child behavior checklist (CBCL) composite raw scores (*N* = 204). Median (IQR) for internalizing, externalizing, and total problems scale, respectively: 6.0 (9.0), 8.0 (12.0), 24.0 (29.0)

Distributions of measured OPE metabolite concentrations are illustrated in Table 2. Median concentrations of BDCIPP (1.26 ng/mL) and DPHP (0.83 ng/mL) were higher than the other OPE metabolites investigated. Detection frequencies were greater than 60% for DPHP, DNBP + DIBP, BDCIPP, BCEP, and BBOEP and ranged between 26.0% and 51.5% for DPRP, BEHP, BMPP, and BCIPP. As shown in Fig. 2, urinary OPE metabolites were weakly correlated with one another (Spearman $\rho$= 0.01–0.27), with DPHP and BDCIPP having the highest correlation among all other OPE metabolites (Spearman $\rho$ =0.27). CBCL distributions among this sample of participants were approximately right skewed, with a median raw score of 6.0 (IQR: 9.0) for the internalizing problems scale, 8.0 (IQR: 12.0) for the externalizing problems scale, and 24.0 (IQR: 29.0) for the total problems scale (see Fig. 3). Approximately 15.2% of participants had internalizing t-scores in the borderline to clinical range (borderline: 6.4%; clinical: 8.8%), 10.3% had externalizing t-scores in the borderline to clinical range (borderline: 4.9%; clinical: 5.4%), and 14.7% had total problems t-scores in the borderline to clinical range (borderline: 4.4%; clinical: 10.3%).

**Table 2:** Distribution of specific gravity adjusted OPE concentrations (ng/mL) in maternal urine (*N* = 204)

Metabolite	Percentiles			Distributions			
	25th	50th	75th	Min–Max	Geometric Mean	Detect Frequency	LOD (ng/mL)
DPHP	0.47	0.83	1.47	0.12–25.59	0.89	99.51%	0.0281
DNBP + DIBP	0.12	0.17	0.25	ND-1.78	0.18	96.57%	0.0441
BDCIPP	0.61	1.26	2.14	ND-34.94	1.05	95.10%	0.0174
BCEP	0.02	0.47	1.60	ND-168.00	0.31	68.63%	0.0200
BBOEP	0.02	0.04	0.08	ND-0.74	0.04	63.24%	0.0199
BCIPP	ND	0.12	0.71	ND-19.90	0.11	51.47%	0.0204
BMPP	ND	0.01	0.04	ND-0.47	0.02	39.71%	0.0115
BEHP	ND	ND	0.03	ND-3.48	0.03	25.00%	0.0170
DPRP	ND	ND	0.06	ND-2.85	0.04	25.98%	0.0278

*OPE* Organophosphate Esters, *LOD* Limit of Detection, *ND* Non-detect, *DPHP* Diphenyl phosphate, *DNBP* + *DIBP* Sum of Di-n-butyl phosphate and Di-isobutyl phosphate, *BDCIPP* Bis(1,3-dichloro-2-propyl) phosphate, *BCEP* Bis(2-chloroethyl) phosphate, *BBOEP* Bis(butoxethyl) phosphate, *BCIPP* Bis(1-chloro-2-propyl) phosphate, *BMPP* Bis(2-methylphenyl) phosphate, *BEHP* Bis(2-ethylhexyl) phosphate, *DPRP* Dipropyl phosphate, *Min* Minimum, *Max* Maximum

### Individual metabolite associations

The unadjusted and adjusted associations between third trimester urinary OPE metabolites and children’s 36-month CBCL raw composite scores are shown in Table 3. Overall, higher concentrations of OPE metabolites were associated with higher externalizing, internalizing, and total problems scores in single metabolite adjusted models. When compared to non-detectable levels of BMPP metabolites, maternal participants with detectable levels of BMPP exposure during the third trimester of pregnancy had children with significantly higher externalizing (*β* = 1.42, 95% CI = 1.04, 1.96) and total problems scores (*β* = 1.35, 95% CI = 1.03, 1.78) after adjustment for key covariates. Maternal participants in the second tertile of BBOEP levels during pregnancy had children with higher externalizing scores (*β* = 1.43, 95% CI = 0.99, 2.09) when compared to maternal participants in the first tertile of BBOEP. However, children’s externalizing scores did not differ for levels in the third compared with first tertile of BBOEP, suggesting possible non-linear effects for this metabolite. There was a marginal association between maternal participants with detectable levels of BMPP concentrations and children with higher internalizing scores (*β* = 1.45, 95% CI: 0.98, 2.14), relative to maternal participants with non-detectable BMPP levels. There were statistically significant interactions between prenatal levels of BCIPP and child sex for both internalizing scores (*p* = 0.02) and total problems scores (*p* = 0.03), and sex-specific associations observed in sex-stratified models. Among male children, internalizing scores (*β* = 2.20, 95% CI: 1.23, 3.95) and total problem scores (*β* = 1.57, 95% CI: 1.06, 2.34) were higher for those with maternal metabolite levels in the third tertile of BCIPP compared with the first tertile (Table S4). This association was not observed among female children between internalizing scores (*β* = 0.61, 9%% CI: 0.30, 1.25) and total problems scores (*β* = 0.69, 95% CI: 0.40, 1.17) and those with maternal metabolite levels in the third tertile of BCIPP, relative to the first tertile (Table S4). In female stratified models, detectable BMPP levels were associated with higher externalizing scores among female children relative to the first tertile (*β* = 1.69, 95% CI: 1.05, 2.70). However, the highest tertile of maternal BBOEP levels during pregnancy were associated with lower total problems among female children (*β* = 0.55, 95% CI: 0.32, 0.94) relative to the first tertile, but the pattern observed between the second tertile versus first tertile of BBOEP suggested increased total problems among female children, suggesting potential non-linear associations. We did not find any statistically significant associations for DPHP, DNBP + DIBP, BDCIPP, BCEP, BEHP, and DPRP with our three CBCL outcomes. However, the pattern of effects were generally suggestive of more linear patterns, with higher internalizing, externalizing, and total problems among children with mothers in the highest tertile of OPE metabolite concentrations compared with the lowest tertile.

**Table 3:** Individual associations between third trimester urinary OPE metabolites (ng/mL) and CBCL raw composite scores (*N* = 204)

	Internalizing		Externalizing		Total Problems	
	Unadjusted *β* (95% CI)	Adjusted^a^ *β* (95% CI)	Unadjusted *β *(95% CI)	Adjusted^a^ *β* (95% CI)	Unadjusted *β* (95% CI)	Adjusted^a^ *β* (95% CI)
DPHP						
T1 (< 0.55)	REF	REF	REF	REF	REF	REF
T2 (0.55–1.15)	0.82 (0.52, 1.31)	0.89 (0.56, 1.41)	0.86 (0.59, 1.26)	0.87 (0.59, 1.26)	0.85 (0.61, 1.17)	0.87 (0.63, 1.20)
T3 (≥ 1.15)	1.04 (0.65, 1.65)	1.03 (0.64, 1.64)	1.10 (0.75, 1.60)	1.01 (0.68, 1.48)	1.10 (0.79, 1.52)	1.04 (0.75, 1.44)
DNBP + DIBP						
T1 (< 0.14)	REF	REF	REF	REF	REF	REF
T2 (0.14–0.21)	1.01 (0.64, 1.61)	1.06 (0.66, 1.69)	1.09 (0.74, 1.60)	1.04 (0.71, 1.52)	1.00 (0.72, 1.38)	0.97 (0.70, 1.35)
T3 (≥ 0.21)	1.06 (0.66, 1.68)	1.18 (0.74, 1.88)	1.13 (0.77, 1.66)	1.15 (0.78, 1.68)	1.04 (0.75, 1.45)	1.09 (0.78, 1.50)
BDCIPP						
T1 (< 0.85)	REF	REF	REF	REF	REF	REF
T2 (0.85–1.83)	0.99 (0.62, 1.57)	1.01 (0.62, 1.64)	1.04 (0.71, 1.52)	0.99 (0.66, 1.47)	1.05 (0.75, 1.45)	1.02 (0.72, 1.43)
T3 (≥ 1.83)	0.86 (0.54, 1.37)	0.97 (0.59, 1.61)	1.15 (0.78, 1.68)	1.16 (0.77, 1.75)	1.07 (0.77, 1.48)	1.12 (0.79, 1.59)
BCEP						
T1 (Non-detect)	REF	REF	REF	REF	REF	REF
T2 (0.04–0.97)	1.10 (0.69, 1.75)	1.19 (0.75, 1.90)	1.06 (0.72, 1.55)	1.14 (0.78, 1.68)	1.00 (0.72, 1.39)	1.04 (0.75, 1.44)
T3 (≥ 0.97)	1.06 (0.66, 1.69)	1.20 (0.74, 1.93)	1.03 (0.70, 1.51)	1.12 (0.75, 1.66)	1.04 (0.75, 1.44)	1.09 (0.78, 1.53)
BBOEP						
T1 (Non-detect)	REF	REF	REF	REF	REF	REF
T2 (0.01–0.06)	1.32 (0.83, 2.08)	1.25 (0.79, 1.98)	1.52 (1.05–2.21)	1.43 (0.99, 2.09)	1.34 (0.97, 1.84)	1.26 (0.92, 1.74)
T3 (≥ 0.06)	0.88 (0.56, 1.38)	0.78 (0.48, 1.25)	0.97 (0.67, 1.41)	0.87 (0.59, 1.29)	0.93 (0.67, 1.27)	0.84 (0.60, 1.17)
BCIPP						
T1 (Non-detect)	REF	REF	REF	REF	REF	REF
T2 (0.03- 0.66)	0.78 (0.49, 1.23)	0.72 (0.46, 1.15)	0.90 (0.62, 1.32)	0.89 (0.61, 1.31)	0.87 (0.63, 1.20)	0.85 (0.62, 1.18)
T3 (≥ 0.66)	1.32 (0.84, 2.09)	1.47 (0.93, 2.34)	1.36 (0.93, 1.98)	1.33 (0.99, 1.95)	1.22 (0.88, 1.68)	1.21 (0.87, 1.67)
BMPP						
Non-detect	REF	REF	REF	REF	REF	REF
Detect	1.42 (0.97, 2.08)	1.45 (0.98, 2.14)	1.39 (1.01, 1.90)	1.42 (1.04, 1.96)	1.34 (1.02, 1.75)	1.35 (1.03, 1.78)
BEHP						
Non-detect	REF	REF	REF	REF	REF	REF
Detect	1.21 (0.78, 1.87)	1.13 (0.73, 1.76)	1.08 (0.75, 1.55)	1.01 (0.77, 1.45)	1.15 (0.85, 1.56)	1.10 (0.81, 1.50)
DPRP						
Non-detect	REF	REF	REF	REF	REF	REF
Detect	0.84 (0.55, 1.29)	0.85 (0.56, 1.31)	1.08 (0.75, 1.53)	1.10 (0.77, 1.56)	0.96 (0.71, 1.30)	0.97 (0.72, 1.31)

All $\beta {\prime}s$ have been exponentiated for interpretation

*OPE* Organophosphate Ester, *T1* Tertile 1, *T2* Tertile 2, *T3* Tertile 3, *CBCL* Child Behavior Checklist, *DPHP* Diphenyl phosphate, *DNBP* + *DIBP* Sum of Di-n-butyl phosphate and Di-isobutyl phosphate, *BDCIPP* Bis(1,3-dichloro-2-propyl) phosphate, *BCEP* Bis(2- chloroethyl) phosphate, *BBOEP* Bis(butoxethyl) phosphate, *BCIPP* Bis(1-chloro-2-propyl) phosphate, *BMPP* Bis(2-methylphenyl) phosphate, *BEHP* Bis(2-ethylhexyl) phosphate, *DPRP* Dipropyl phosphate, *GA* Gestational age, *BMI* Body Mass Index

^a^Model adjusted for recruitment site, maternal age, race/ethnicity, household annual income, education, pre-pregnancy BMI, GA at sample collection, child adjusted age at CBCL administration, season, infant birth order, child sex

**Fig. 4:**
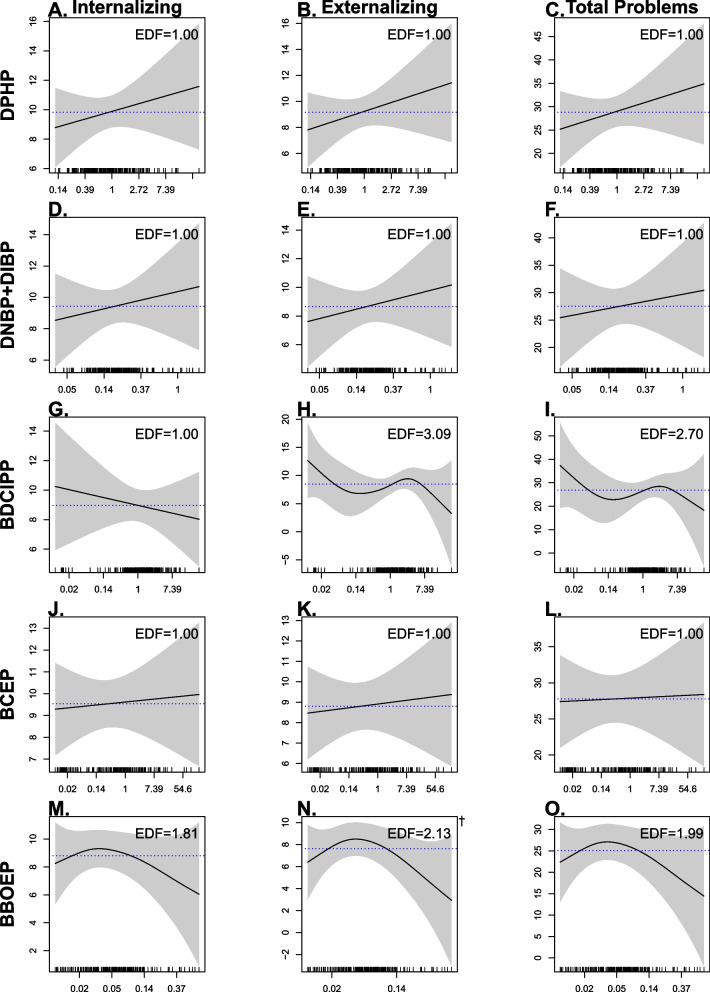
Associations between urinary prenatal OPE metabolite concentrations (ng/mL) and CBCL composite raw scores, using generalized additive models (*N* = 204). Note: All models adjusted for recruitment site, maternal age, race/ethnicity, household annual income, education, pre-pregnancy BMI, GA at sample collection, child adjusted age at CBCL administration, season, infant birth order, child sex. OPE, Organophosphate Ester; CBCL, Child Behavior Checklist; DPHP, Diphenyl phosphate; DNBP + DIBP, Sum of Di-n-butyl phosphate and Di-isobutyl phosphate; BDCIPP, Bis(1,3-dichloro-2-propyl) phosphate; BCEP, Bis(2-chloroethyl) phosphate; BBOEP, Bis(butoxethyl) phosphate. †Significant non-linearity

When compared to the linear regression model, we found evidence of a better model fit using GAMs for the association between prenatal BBOEP concentrations and children’s externalizing score at 36 months (*p* = 0.04), with higher children’s externalizing scores at moderate concentrations of prenatal BBOEP but lower children’s externalizing scores at lower and higher concentrations of prenatal BBOEP (see Fig. 4n). However, associations between prenatal urinary OPE metabolites and CBCL raw composite scores were not statistically significant when using GAMs, nor were interactions and sex-specific smooths between each OPE metabolite and child sex (Figs. S2-S4).

In sensitivity analyses evaluating CBCL t-scores as an alternative parametrization of CBCL scores, associations between maternal BMPP during pregnancy and children’s internalizing and externalizing t-scores were consistent with the associations observed when using the CBCL raw scores (see Table S5). Similar to our findings with raw scores, when compared to maternal participants in the first tertile of BBOEP exposure, maternal participants in the second tertile of BBOEP exposure had children with significantly higher externalizing (*β* = 1.08, 95% CI = 1.00, 1.17) and total problem (*β* = 1.09, 95% CI = 1.00, 1.18) t-scores, but results for the third tertile were not statistically significant across the internalizing, externalizing, or total problems scales. Results between OPE metabolites and CBCL composite t-scores in GAMs remained consistent with the patterns observed between OPE metabolites and CBCL composite raw scores (see Fig. S5). In a sensitivity analysis excluding maternal participants who reported smoking during pregnancy, associations were consistent with the full study sample, both for linear regression models (see Table S6) and GAMs (see Fig. S6).

### Mixtures associations

**Fig. 5:**
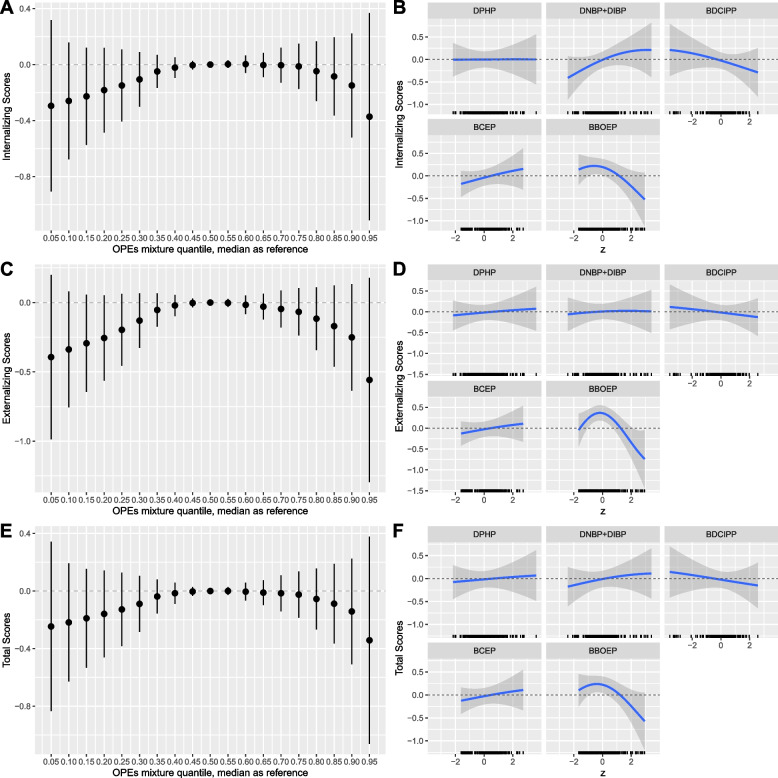
Prenatal OPE urinary metabolite mixtures (ng/mL) and CBCL composite raw scores, using BKMR (*N* = 204). Figure 5 includes: 1) the estimated difference in CBCL composite score when setting all metabolites to the percentile specified on the x-axis compared with setting all metabolites to their median values (column 1), 2) the univariate relationship between each metabolite and CBCL outcome, while other metabolites are fixed at their medians, and a rug plot showing the distribution of the specified metabolite along the x-axis of each panel (column 2). All models were adjusted for recruitment site, maternal age, race/ethnicity, household annual income, education, pre-pregnancy BMI, GA at sample collection, child adjusted age at CBCL administration, season, infant birth order, child sex. OPE metabolites and CBCL raw scores were natural log-transformed, mean centered, and standard deviation scaled. Continuous covariates were mean-centered and standard deviation scaled. Note: BKMR, Bayesian Kernel Machine Regression; OPE, Organophosphate Ester; CBCL, Child Behavior Checklist; DPHP, Diphenyl phosphate; DNBP + DIBP, Sum of Di-n-butyl phosphate and Di-isobutyl phosphate; BDCIPP, Bis(1,3-dichloro-2-propyl) phosphate; BCEP, Bis(2-chloroethyl) phosphate; BBOEP, Bis(butoxethyl) phosphate

Concurrent increases in concentrations of all metabolites with CBCL composite raw scores had a non-monotonic, inverted U-shaped pattern, with lower CBCL composite scores at both higher and lower quantiles of metabolite mixtures when compared to the median. However, since all 95% CrI crossed 0, there were no cumulative associations between the overall OPE metabolite mixture and the internalizing, externalizing, and total problems raw scores (see Fig. 5A, C, and E).

Relationships between each individual metabolite, while fixing other metabolites at their median values, and children’s internalizing, externalizing, and total problems scores adjusting for key covariates are shown in Fig. 5B, D, and F. A marginal association was observed between prenatal DNBP + DIBP and the internalizing problems scale, with an increase in DNBP + DIBP from the 25^th^ to the 75^th^ percentile associated with a 0.15 (95% CrI: -0.02, 0.32) standard deviation increase on the internalizing problems scale, when all other metabolites were fixed at their median values and after adjustment for key covariates (Table 4). The association between BBOEP and each CBCL composite raw score was consistently non-linear and an inverted U-shaped, with higher internalizing, externalizing, and total problems scores among children at moderate concentrations of BBOEP but lower CBCL composite scores at lower and higher BBOEP concentrations. The associations between DNBP + DIBP and children’s total problems scores were positive and linear. However, the association between DNBP + DIBP and the externalizing score was relatively null. The shape and direction between BDCIPP, BCEP, and BBOEP and each CBCL composite raw score were consistent across scales; we observed an inverse, linear association with BDCIPP and each CBCL raw score and a positive and linear association between BCEP and each CBCL composite raw score. We found a relatively null association between DPHP and internalizing, externalizing, and total problems raw scores. Effect estimates evaluating the difference in CBCL composite raw scores for a change in the specified metabolite from the 25^th^ the 75th percentile, holding all other metabolites in the mixture at their median values and adjusting for key covariates, had 95% CrIs which spanned 0 (Table 4).

**Table 4:** Posterior inclusion probabilities (PIPs) and single exposure effect estimates for each prenatal OPE metabolite in the Bayesian kernel machine regression (BKMR) mixture and CBCL composite raw score

Metabolite	PIPs	Effect Estimates	95% Credible Interval
Internalizing Scale			
DPHP	0.27	0.002	-0.17, 0.18
DNBP + DIBP	0.53	0.15	-0.02, 0.32
BDCIPP	0.34	-0.09	-0.24, 0.06
BCEP	0.37	0.15	-0.10, 0.41
BBOEP	**0.64**^**a**^	-0.10	-0.33, 0.14
Externalizing Scale			
DPHP	0.15	0.04	-0.13, 0.20
DNBP + DIBP	0.19	0.02	-0.13, 0.17
BDCIPP	0.15	-0.04	-0.17, 0.10
BCEP	0.20	0.11	-0.14, 0.36
BBOEP	**0.81**^**a**^	-0.02	-0.28, 0.24
Total Problems Scale			
DPHP	0.24	0.03	-0.14, 0.21
DNBP + DIBP	0.30	0.06	-0.10, 0.22
BDCIPP	0.26	-0.04	-0.19, 0.10
BCEP	0.28	0.11	-0.15, 0.36
BBOEP	**0.59**^**a**^	-0.07	-0.31, 0.17

Effect estimates reflect the difference in CBCL composite score for a change in the specified metabolite from the 25th to 75th percentile, holding all other metabolites in the mixture at their median values and adjusting for recruitment site, maternal age, race/ethnicity, household annual income, education, pre-pregnancy BMI, GA at sample collection, child adjusted age at CBCL administration, season, infant birth order, child sex

*OPE* Organophosphate Ester, *CBCL* Child Behavior Checklist, *DPHP* Diphenyl phosphate, *DNBP* + *DIBP* Sum of Di-n-butyl phosphate and Di-isobutyl phosphate, *BDCIPP* Bis(1,3-dichloro-2-propyl) phosphate, *BCEP* Bis(2-chloroethyl) phosphate, *BBOEP* Bis(butoxethyl) phosphate

^a^Highest PIPs value

**Fig. 6:**
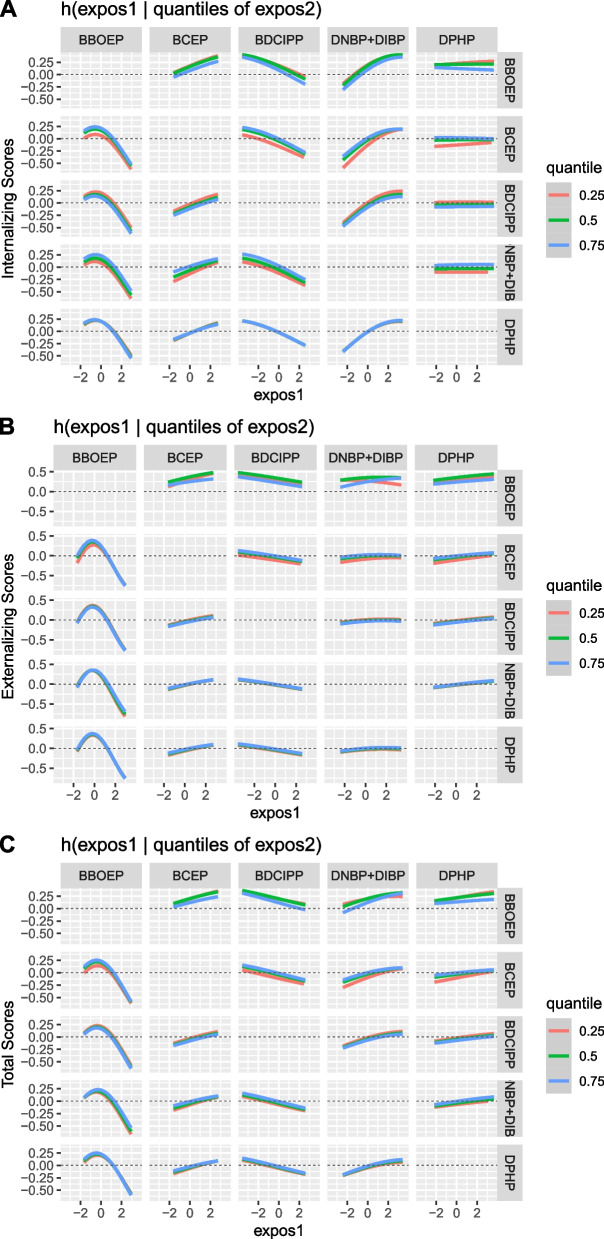
Bivariate associations between prenatal OPE urinary metabolite mixtures (ng/mL) and CBCL composite raw scores, using BKMR (*N* = 204). Figure 6 shows the bivariate association between each OPE metabolite (labelled in the column) and CBCL composite score (Y axis), while setting a second metabolite (labelled in the row) to its 25^th^, 50^th^, and 75^th^ percentile and all other metabolites to their median. All models were adjusted for recruitment site, maternal age, race/ethnicity, household annual income, education, pre-pregnancy BMI, GA at sample collection, child adjusted age at CBCL administration, season, infant birth order, child sex. OPE metabolites and CBCL raw scores were natural log-transformed, mean centered, and standard deviation scaled. Continuous covariates were mean-centered and standard deviation scaled. Note: BKMR, Bayesian Kernel Machine Regression; OPE, Organophosphate Ester; CBCL, Child Behavior Checklist; DPHP, Diphenyl phosphate; DNBP + DIBP, Sum of Di-n-butyl phosphate and Di-isobutyl phosphate; BDCIPP, Bis(1,3-dichloro-2-propyl) phosphate; BCEP, Bis(2-chloroethyl) phosphate; BBOEP, Bis(butoxethyl) phosphate. Possible interactions were visually identified between the following metabolites for: internalizing scores (BDCIPP and BBOEP, DNBP + DIBP and BBOEP, DPHP and BBOEP, DNBP + DIBP and BCEP, DPHP and BCEP, BCEP and DNBP + DIBP, and DNBP + DIBP and BDCIPP), externalizing scores (BCEP and BBOEP, BDCIPP and BBOEP, DNBP + DIBP and BBOEP, and DPHP and BBOEP), and total problems scores (BCEP and BBOEP, BDCIPP and BBOEP, DNBP + DIBP and BBOEP, DPHP and BBOEP, DNBP + DIBP and BCEP, DPHP and BCEP, BCEP and DNBP + DIBP, BCEP and DPHP, and DNBP + DIBP and DPHP)

Possible pairwise interactions between OPE metabolites and CBCL composite raw scores were visually identified using BKMR (Fig. 6A, B and C). PIPs for each pairwise interaction were also estimated using the NLinteraction method (Fig. S7) and pairwise interactions with the highest ranked PIPs further examined [52]. In the internalizing scores model, the interaction between DNBP + DIBP and BCEP had the highest pairwise PIP estimated using NLinteraction (Fig. S7). With BKMR, we observed a stronger positive association between DNBP + DIBP and internalizing scores at higher quartiles of BCEP. Within the externalizing scores model, the highest interaction PIP from NLinteraction was observed for DNBP + DIBP and BBOEP. With BKMR, we observed a positive association between DNBP + DIBP and externalizing scores at the 50^th^ and 75^th^ percentile of BBOEP, but an inverse association between DNBP + DIBP and externalizing scores at the 25^th^ percentile of BBOEP. In the total problems scores model, the largest interaction PIP identified by NLinteraction was for DNBP + DIBP and BCEP. With BKMR, we observed a stronger positive association between DNBP + DIBP and total problems scores at higher quartiles of BCEP.

Finally, the highest ranked interaction PIP for each CBCL composite score was further explored using GAMs to evaluate interaction *p*-values. We found a statistically significant interaction between DNBP + DIBP and total problems scores by BCEP concentrations modeled both continuously (*p* = 0.03) and in tertiles (*p* = 0.049), providing suggestive evidence of a potential interaction. Associations between prenatal DNBP + DIBP and children’s total problems scores by tertiles of BCEP were generally consistent with those observed in bivariate mixtures plots (see Fig. S8).

We found consistent results in sensitivity analyses evaluating CBCL t-scores as an alternative parametrization of the CBCL raw scores and as well as when we excluded mothers who reported smoking during pregnancy in our mixtures models (see Figs. S9 and S10). Results from models assuming a lower degree of smoothness (b = 50) were very similar to primary results (Fig. S11). However, the results for models assuming a higher degree of smoothness (b = 1000) had a more linear pattern which appeared null in both the internalizing and total problems cumulative mixtures plots but maintained the inverted U-shaped pattern for the externalizing problems cumulative mixtures plots (Fig. S12). Rankings of interaction PIPs from NLinteraction were consistent when increasing the value of the threshold parameter (Fig. S13). Similarly, there were consistent results between models evaluating OPE mixtures among metabolites with a detect frequency > 80% (DPHP, DNBP + DIBP, BDCIPP) and neurobehavioral outcomes and the primary analysis (Figs. S14 and S15).

## Discussion

In this study of 204 predominately Hispanic and low-income mother-child dyads living in Los Angeles, California, we found important associations between independent OPE metabolites and neurobehavioral outcomes at 36 months of age as well as evidence for interacting effects between OPE metabolites and child’s sex. In single OPE analyses, detectable urinary BMPP concentrations during the third trimester of pregnancy were associated with higher internalizing problems, externalizing problems, and total problems in children at 36 months of age relative to those with non-detectable prenatal levels of BMPP. We also found that middle tertile (0.01–0.06 ng/mL) but not highest tertile (> 0.06 ng/mL) concentrations of urinary BBOEP during pregnancy were associated with higher externalizing problems scores in children at 36 months of age when compared to those with non-detectable prenatal levels of BBOEP. Statistically significant non-linear and U-shaped patterns were observed between prenatal maternal BBOEP concentrations and children’s externalizing scores, with higher scores observed in the second tertile of BBOEP concentrations. Statistically significant interactions between BCIPP exposure and child’s sex were also identified for internalizing and total problems outcome models, with higher internalizing and total problems scores observed for male children whose mothers fell in the highest tertile of BCIPP compared with male children whose mothers fell in the lowest tertile of BCIPP. There were also higher externalizing scores among female children whose mothers had detectable BMPP levels relative to non-detectable levels. However, maternal participants in the third tertile of BBOEP concentrations had female children with lower total problems relative to the first tertile but patterns of increased total problems with the second tertile of BBOEP exposure, suggestive of potential non-monotonic BBOEP impacts on neurobehavior.

Although we did not observe an overall association between the mixture of DPHP, DNBP + DIBP, BDCIPP, BCEP, and BBOEP and neurobehavioral outcomes at 36-months, we did observe a positive association between prenatal DNBP + DIBP concentration and children’s internalizing problems, when fixing BDCIPP, BCEP, BBOEP and DPHP at their median concentrations. We also found evidence of a potential interaction between prenatal DNBP + DIBP and BCEP concentrations for total problems. Overall, our results suggest adverse effects of OPE exposures on neurobehavioral development, specifically for OPE metabolites commonly understudied and under monitored in pregnant individuals, with non-linear patterns and sex-specific interactions suggestive of endocrine-disrupting effects.

Limited epidemiological evidence has reported adverse associations between prenatal OPE exposures and neurobehavioral outcomes in early childhood. The Pregnancy, Infection, and Nutrition (PIN) Study, a prospective birth cohort of predominately non-Hispanic white (~ 82%) and college educated individuals in North Carolina, found positive associations between BDCIPP and DPHP concentrations in prenatal urine and behavioral symptoms and externalizing problems using the Behavioral Assessment System for Children (BASC-2) among 199 children at 36 months of age [38]. The PIN study also reported an inverse association between isopropyl-phenyl phenyl phosphate (ip-PPP) and internalizing problems. The CHAMACOS study, a pregnancy cohort of predominately low-income and Hispanic participants in Central California found increased hyperactivity, using the BASC-2 at 7 years of age, with maternal urinary ip-PPP concentrations during pregnancy [39]. Another study by Choi et al., found a higher risk of ADHD among children with greater than median exposure to DPHP during pregnancy for participants in the Norwegian mother, father, and child cohort study (MoBa), with more pronounced associations among girls and a decreased risk for ADHD with decreasing joint exposure to OPE metabolites, including DPHP and DNBP, and phthalates [53]. Other studies evaluating early life OPE exposures in dust concentrations on neurobehavioral outcomes have found similar adverse impacts between the summed exposure of OPEs (TDCPP, TPP, TCPP, and TCEP) and less responsible behavior and externalizing behavior problems using the teacher-rated Social Skills Improvement Rating Scale (SSIS) [54]. Similarly, early exposures to TCEP in household dust have been associated with higher externalizing problems and early exposures to bisphenol A bis (diphenylphosphate) (BPA-BDPP) and resorcinol bis (diphenylphosphate) (PBDPP) in household dust have been associated with higher externalizing and internalizing problems at 18 months using the CBCL [40].

In our study, we did not observe statistically significant associations between BDCIPP and DPHP and externalizing symptoms, although the pattern for DPHP and externalizing symptoms in single metabolite models showed a similar direction of effect to prior literature. However, we observed adverse associations between detectable prenatal BMPP levels and higher internalizing, externalizing, and total problems and BBOEP concentrations and higher externalizing scores in single metabolite analyses. Additionally, positive associations between the highest tertile of BCIPP levels and male children’s internalizing and total problems scores were found, along with positive associations between detectable BMPP and female children’s externalizing problems and negative associations between the third tertile of BBOEP and female children’s total problems. We also observed a marginal association between DNBP + DIBP and the internalizing problems scale when accounting for the rest of the mixture. Discrepancies in results across each of these studies may be attributable to a variety of factors, including but not limited to, heterogenous participant characteristics and exposure distributions (Table S7), differences in the timing of exposure measurements (mid vs late gestation and varying years), outcome measurements, and children’s ages at behavioral assessments. For instance, the PIN study had higher median concentrations of DPHP (1.38 ng/mL vs. 0.83 ng/mL) and BDCIPP (2.01 ng/mL vs. 1.26 ng/mL) compared to MADRES participants; median concentrations among the CHAMACOS participants were relatively similar to those of MADRES for DPHP (0.93 ng/mL vs. 0.83 ng/mL) but lower for BDCIPP (0.41 ng/mL vs. 1.26 ng/mL). Participants in the MoBa cohort study had much lower median concentrations of DPHP (0.45 ng/mL vs. 0.83 ng/mL), BBOEP (0.07 ng/mL vs. 0.04), and BDCIPP (< 0.17 vs 1.26 ng/mL) compared to participants in the MADRES study. Additionally, the PIN study measured OPEs between 24–29 weeks’ gestation between 2001–2005, CHAMACOS at a mean gestational age of 26 weeks between 1999–2000, and MoBa at 17 weeks from 1999–2008, compared to MADRES at a mean gestational age of 31 weeks from 2017–2019. The age at which children’s neurobehavioral development was assessed and the instruments used to measure neurobehavioral development also differed across these studies. While CHAMACOS assessed hyperactivity and attention problems using the BASC-2 when children were approximately 7 years old, the PIN study used all BASC-2 scales to evaluate neurobehavioral outcomes when children were 36 months of age. The MoBa study used the Norwegian Patient Registry to identify clinically diagnosed ADHD for children age 2.5 to 10 years. Despite these discrepancies across studies, the epidemiological literature generally suggests adverse impacts of OPE metabolites on neurobehavioral development.

Emerging toxicological and epidemiological evidence suggests several mechanisms which may underlie the adverse association between prenatal exposures to environmentally relevant doses of OPEs and early behavioral and emotional development. Hypothesized mechanisms include direct impacts of prenatal OPEs on the neurological morphology and functioning of important neurobehavioral structures, including perturbations of glutamate and GABA neurotransmitters [36, 55–60], inflammation [58, 61], glia activation [62, 56], oxidative stress [58, 36, 63], and decreased neuronal growth and network activity [55, 64–66]. For instance, in an in vitro model study using 3D rat primary neural organotypic, three OPEs, including TMPP, were associated with decreased GABA, glutamate, and dopamine neurotransmitters, along with evidence of possible inflammatory response and interference of myelination [56]. Furthermore, in animal studies using Wistar rats, the placenta has been implicated as a potentially important mechanism of developmental neurotoxicity from prenatal OPE exposures, with higher OPE accumulation in placental tissue among male placentas and further evidence of reduced forebrain serotonin (5-HT) and endocrine disruption, inflammation, and altered neurotransmitter production in the placenta [67–70]. Additional hypothesized mechanisms include maternal-mediated impacts of prenatal OPEs on early neurobehavior via critical mechanisms for neurobehavioral development, such as endocrine-disrupting pathways, which play a vital role in the development of the brain structures and processes important to behavior and which may be sex-specific [71]. Prior epidemiological studies have found an association between OPE exposures and altered levels of thyroid stimulating hormone (TSH) [72] and disruption of other thyroid hormones [73], along with disruption of sex-steroid hormones and sex-steroid binding globulins [74]. Given the rapid development of neurological systems during pregnancy, low-level chronic exposure to OPEs during pregnancy may exert neurotoxic effects on the developing fetus, with long-lasting neurobehavioral implications [37, 38].

This study has several important strengths. Its prospective design provided us with the opportunity to collect urine samples during potentially sensitive periods (i.e., pregnancy) to measure OPEs prior to our outcome of interest. An additional strength of this study was the use of prenatal urinary metabolites as a measure of in utero exposure to OPEs, given that maternal urinary OPE metabolites are considered reliable indicators of potential fetal OPE exposures [15]. We also measured various previously understudied OPE metabolites, including DNBP + DIBP, BCIPP, BCEP, BBOEP, DRPR, BMPP, and BEHP, which advances opportunities for risk assessment and subsequent interventions. Furthermore, the population evaluated in this study was largely comprised of pregnant individuals of Latin American origin, who are historically underrepresented in U.S. biomedical and population health research and disproportionally burdened by environmental exposures [75], providing us with the opportunity to inform environmental justice solutions. An additional strength of this study is the use of a flexible environmental mixture modeling approach to assess the association between mixtures of OPE metabolites and neurobehavioral outcomes at 36 months.

However, our study also has some limitations. Since single spot urine samples collected during the third trimester were used to assess OPE exposures throughout pregnancy, there may have been some exposure misclassification. However, previous studies indicate moderate to good reproducibility for DPHP and BDCIPP levels throughout pregnancy [76, 77]. Additionally, although many key covariates identified in the literature were adjusted for, residual confounding could still be present, especially for postnatal OPE exposures, which could impact neurobehavioral outcomes. The relatively modest analytical sample analyzed in this study is another limitation since we may have been underpowered to detect associations between OPE mixtures and neurobehavioral outcomes. Furthermore, although our use of a flexible environmental mixture modeling approach was used to assess joint OPE exposures, we were unable to explore the impacts of joint OPE exposures among metabolites with low detect frequencies, such as BMPP, which we found to adversely impact neurobehavioral development.

## Conclusion

In this prospective pregnancy cohort of predominately low-income and Hispanic pregnant individuals living in Los Angeles, we found adverse associations between prenatal exposures to multiple previously understudied OPEs and children’s neurobehavioral outcomes at 36 months. There was also suggestive evidence of interactions between metabolites, highlighting the importance of evaluating OPEs beyond the effects of a single metabolite, along with non-linear and sex-specific associations between OPEs and children’s neurobehavioral development. Given the scarcity of studies evaluating associations between prenatal OPE metabolites and early neurobehavioral outcomes, additional studies exploring these associations, for exposures during both the prenatal and postnatal periods, are warranted.

### Supplementary Information

**Additional file 1: Supplemental Figure 1.** Directed Acyclic Graph (DAG) of Prenatal OPE metabolites and Child Neurobehavioral Development. **Supplemental Figure 2.** Associations Between Urinary Prenatal OPE Metabolite Concentrations (ng/mL) and Internalizing Scores by Child Sex, Using Generalized Additive Models (*N* = 204). **Supplemental Figure 3.** Associations Between Urinary Prenatal OPE Metabolite Concentrations (ng/mL) and Externalizing Scores by Child Sex, Using Generalized Additive Models (*N* = 204). **Supplemental Figure 4.** Associations Between Urinary Prenatal OPE Metabolite Concentrations (ng/mL) and Total Problems Scores by Child Sex, Using Generalized Additive Models (*N* = 204). **Supplemental Figure 5.** Associations Between Urinary Prenatal OPE Metabolite Concentrations (ng/mL) and CBCL Composite T-Scores, Using Generalized Additive Models (*N* = 204). **Supplemental Figure 6.** Associations Between Urinary Prenatal OPE Metabolite Concentrations (ng/mL) and CBCL Composite Raw Scores Among Participants Who Reported No In-Utero Smoking, Using Generalized Additive Models (*N* = 199). **Supplemental Figure 7.** Posterior Inclusion Probabilities (PIPs) for Pairwise Interactions Between OPE Metabolites and CBCL Composite Raw Scores Using NLinteraction Method. **Supplemental Figure 8.** Prenatal DNBP+DIBP Exposures and Children’s Total Problems Scores by Tertiles of BCEP, Using Generalized Additive Models. **Supplemental Figure 9.** Prenatal OPE Urinary Metabolite Mixtures (ng/mL) and CBCL Composite T-Scores, Using BKMR (*N* = 204). **Supplemental Figure 10.** Prenatal OPE Urinary Metabolite Mixtures (ng/mL) and CBCL Composite Raw Scores Among Participants Who Reported No In-Utero Smoking, Using BKMR (*N* = 199). **Supplemental Figure 11.** Prenatal OPE Urinary Metabolite Mixtures (ng/mL) and CBCL Composite Raw Scores, Using BKMR Varying the Smoothing Parameter to b = 50. **Supplemental Figure 12.** Prenatal OPE Urinary Metabolite Mixtures (ng/mL) and CBCL Composite Raw Scores, Using BKMR Varying the Smoothing Parameter to b = 1000. **Supplemental Figure 13.** Posterior Inclusion Probabilities (PIPs) for Pairwise Interactions Between OPE Metabolites and CBCL Composite Raw Scores Using NLinteraction Method and Increasing the Threshold to 0.25. **Supplemental Figure 14.** Prenatal OPE Urinary Metabolite Mixtures and CBCL Composite Raw Scores, Using BKMR and Metabolites with Detect Frequency >80% Only (*N* = 204). **Supplemental Figure 15.** Bivariate Associations Between Prenatal OPE Urinary Metabolite Mixtures (ng/mL) and CBCL Composite Raw Scores, Using BKMR and Metabolites with Detect Frequency >80% Only (*N* = 204). **Supplemental Table 1.** Parent Compounds of OPE Metabolites Analyzed and Common Applications. **Supplemental Table 2.** Distribution of Specific Gravity Adjusted OPE Concentrations (ng/mL) in Urine for Maternal Participants Analyzed (*N* = 204) vs the Full Sample of Maternal Participants with OPEs Available (*N* = 426). **Supplemental Table 3.** Comparison of Participant Characteristics Analyzed in the Analytical Dataset (*N* = 204) to Subset with OPE Metabolite Concentrations Available (*N* = 426) and Full MADRES Participants Who Delivered Children in the Study by August 28^th^, 2022 (*N* = 774). **Supplemental Table 4.** Individual Associations Between Third Trimester Urinary OPE Metabolites (ng/mL) and CBCL Raw Composite Scores by Child Sex (*N* = 204). **Supplemental Table 5.** Individual Associations Between Third Trimester Urinary OPE Metabolites (ng/mL) and CBCL Composite T-Scores (*N* = 204). **Supplemental Table 6.** Individual Associations Between Third Trimester Urinary OPE Metabolites (ng/mL) and CBCL Raw Composite Scores Among Participants Who Reported No In-Utero Smoking (*N* = 199). **Supplemental Table 7.** Median Concentrations (ng/mL) of Urinary OPE Metabolites Across Published Studies.

## Data Availability

The datasets used and/or analyzed during the current study are available from the corresponding author on reasonable request and after approval by the USC Institutional Review Board.

## Abbreviations

OPEs: Organophosphate Esters
MADRES: Maternal And Developmental Risks from Environmental and Social Stressors
CBCL: Child Behavior Checklist
DPHP: Diphenyl phosphate
DNBP + DIBP: Di-n-butyl phosphate and di-isobutyl phosphate
BDCIPP: Bis(1,3,-dichloro-2-propyl) phosphate
BCEP: Bis(2-chloroethyl) phosphate
BBOEP: Bis(butoxethyl) phosphate
BCIPP: Bis(1-chloro-2-propyl) phosphate
BMPP: Bis(2-methylphenyl) phosphate
BEHP: Bis(2-ethylhexyl) phosphate
DPRP: Dipropyl phosphate
BKMR: Bayesian kernel Machine Regression
PBDEs: Polybrominated diphenyl ethers
DAG: Directed Acyclic Graph
BMI: Body mass index
GA: Gestational age
LOWESS: Locally Weighted Scatterplot Smoothing
GAM: Generalized additive model
CrI: Credible Interval
CI: Confidence Interval
MCMC: Markov chain Monte Carlo
WAIC: Watanabe-Akaike information criterion
PIPs: Posterior Inclusion Probabilities
