# Supplementary material for: Prenatal exposures to organophosphate ester metabolite mixtures and children’s neurobehavioral outcomes in the MADRES pregnancy cohort
Source: Environ Health. 2023 Sep 22;22:66. doi: 10.1186/s12940-023-01017-3 (PMC10515433; doi:10.1186/s12940-023-01017-3)
Supplement: Supplementary file 1 — Additional file 1: Supplemental Figure 1. Directed Acyclic Graph (DAG) of Prenatal OPE metabolites and Child Neurobehavioral Development. Supplemental Figure 2. Associations Between Urinary Prenatal OPE Metabolite Concentrations (ng/mL) and Internalizing Scores by Child Sex, Using Generalized Additive Models (N = 204). Supplemental Figure 3. Associations Between Urinary Prenatal OPE Metabolite Concentrations (ng/mL) and Externalizing Scores by Child Sex, Using Generalized Additive Models (N = 204). Supplemental Figure 4. Associations Between Urinary Prenatal OPE Metabolite Concentrations (ng/mL) and Total Problems Scores by Child Sex, Using Generalized Additive Models (N = 204). Supplemental Figure 5. Associations Between Urinary Prenatal OPE Metabolite Concentrations (ng/mL) and CBCL Composite T-Scores, Using Generalized Additive Models (N = 204). Supplemental Figure 6. Associations Between Urinary Prenatal OPE Metabolite Concentrations (ng/mL) and CBCL Composite Raw Scores Among Participants Who Reported No In-Utero Smoking, Using Generalized Additive Models (N = 199). Supplemental Figure 7. Posterior Inclusion Probabilities (PIPs) for Pairwise Interactions Between OPE Metabolites and CBCL Composite Raw Scores Using NLinteraction Method. Supplemental Figure 8. Prenatal DNBP+DIBP Exposures and Children’s Total Problems Scores by Tertiles of BCEP, Using Generalized Additive Models. Supplemental Figure 9. Prenatal OPE Urinary Metabolite Mixtures (ng/mL) and CBCL Composite T-Scores, Using BKMR (N = 204). Supplemental Figure 10. Prenatal OPE Urinary Metabolite Mixtures (ng/mL) and CBCL Composite Raw Scores Among Participants Who Reported No In-Utero Smoking, Using BKMR (N = 199). Supplemental Figure 11. Prenatal OPE Urinary Metabolite Mixtures (ng/mL) and CBCL Composite Raw Scores, Using BKMR Varying the Smoothing Parameter to b = 50. Supplemental Figure 12. Prenatal OPE Urinary Metabolite Mixtures (ng/mL) and CBCL Composite Raw Scores, Using BKMR Varying the Smoothing [file 12940_2023_1017_MOESM1_ESM.docx]

Supplemental Figure 1: Directed Acyclic Graph (DAG) of Prenatal OPE metabolites and Child Neurobehavioral Development


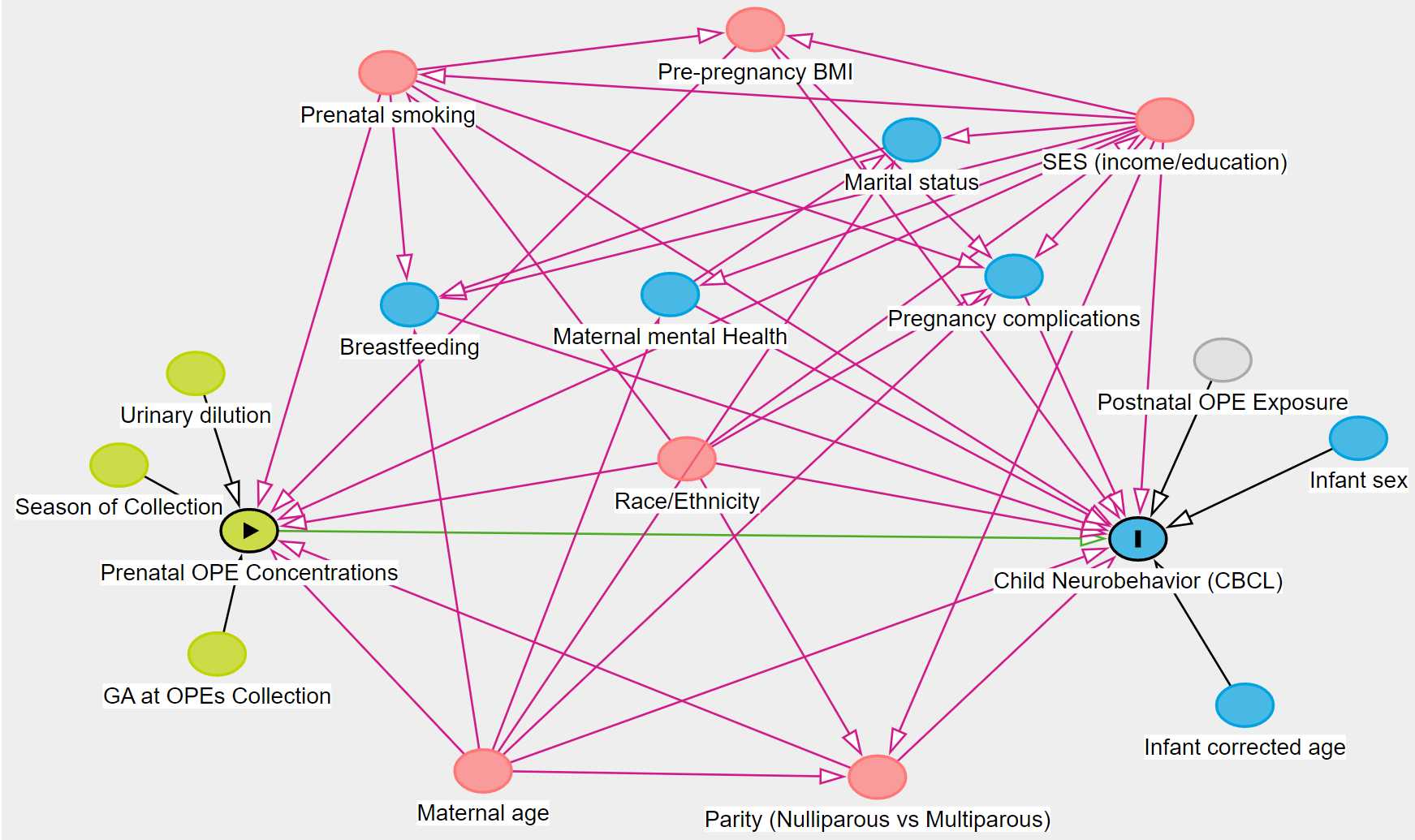


Directed Acyclic Graph (DAG) used to identify potential confounders and precision variables. The DAG was created using DAGitty. Green ovals represent exposures or predictors of the exposure, pink ovals represent potential confounders, and blue ovals represent the outcome or predictors of outcome.

*Minimally sufficient set*: Maternal age, parity, pre-pregnancy BMI, prenatal smoking, race/ethnicity, socioeconomic status (SES)

| Supplemental Figure 2: Associations Between Urinary Prenatal OPE Metabolite Concentrations (ng/mL) and Internalizing Scores by Child Sex, Using Generalized Additive Models (N=204) |
| --- |
| 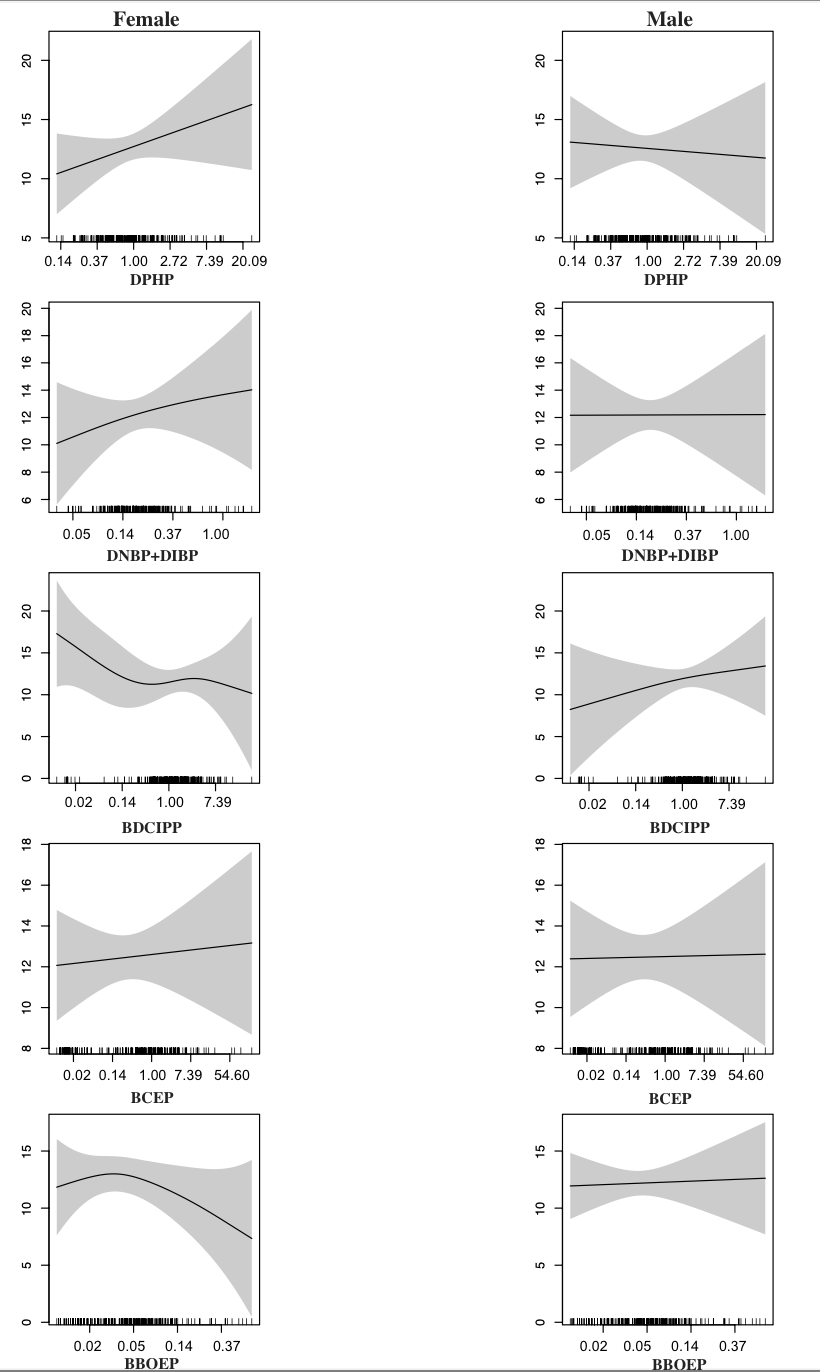  † |
| All models adjusted for recruitment site, maternal age, race/ethnicity, household annual income, education, pre-pregnancy BMI, GA at sample collection, child adjusted age at CBCL administration, season, infant birth order, child sex. Note: OPE, Organophosphate Ester; CBCL, Child Behavior Checklist; DPHP, Diphenyl phosphate; DNBP+DIBP, Sum of Di-n-butyl phosphate and Di-isobutyl phosphate; BDCIPP, Bis(1,3-dichloro-2-propyl) phosphate; BCEP, Bis(2-chloroethyl) phosphate; BBOEP, Bis(butoxethyl) phosphate. |

| Supplemental Figure 3: Associations Between Urinary Prenatal OPE Metabolite Concentrations (ng/mL) and Externalizing Scores by Child Sex, Using Generalized Additive Models (N=204) |
| --- |
| 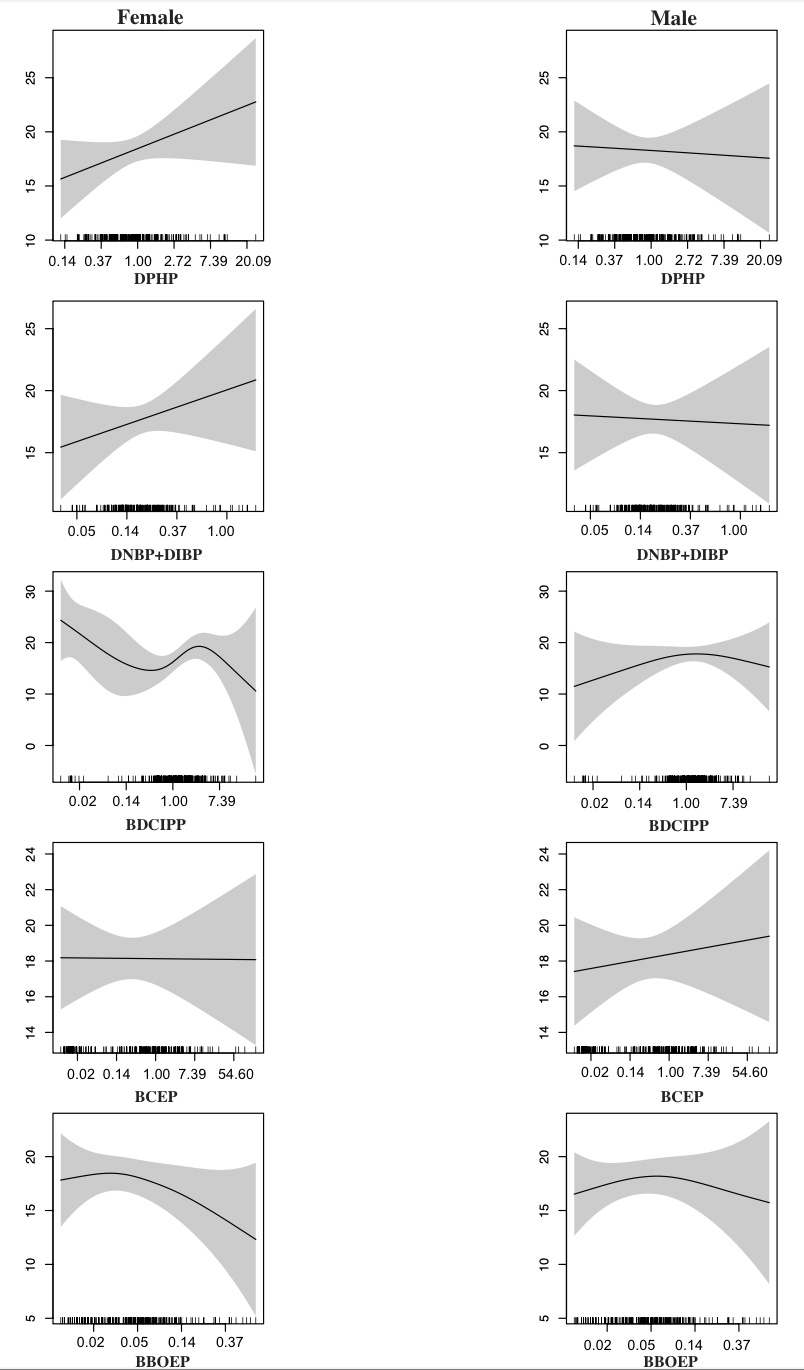 |
| All models adjusted for recruitment site, maternal age, race/ethnicity, household annual income, education, pre-pregnancy BMI, GA at sample collection, child adjusted age at CBCL administration, season, infant birth order, child sex. Note: OPE, Organophosphate Ester; CBCL, Child Behavior Checklist; DPHP, Diphenyl phosphate; DNBP+DIBP, Sum of Di-n-butyl phosphate and Di-isobutyl phosphate; BDCIPP, Bis(1,3-dichloro-2-propyl) phosphate; BCEP, Bis(2-chloroethyl) phosphate; BBOEP, Bis(butoxethyl) phosphate. |

| Supplemental Figure 4: Associations Between Urinary Prenatal OPE Metabolite Concentrations (ng/mL) and Total Problems Scores by Child Sex, Using Generalized Additive Models (N=204) |
| --- |
| 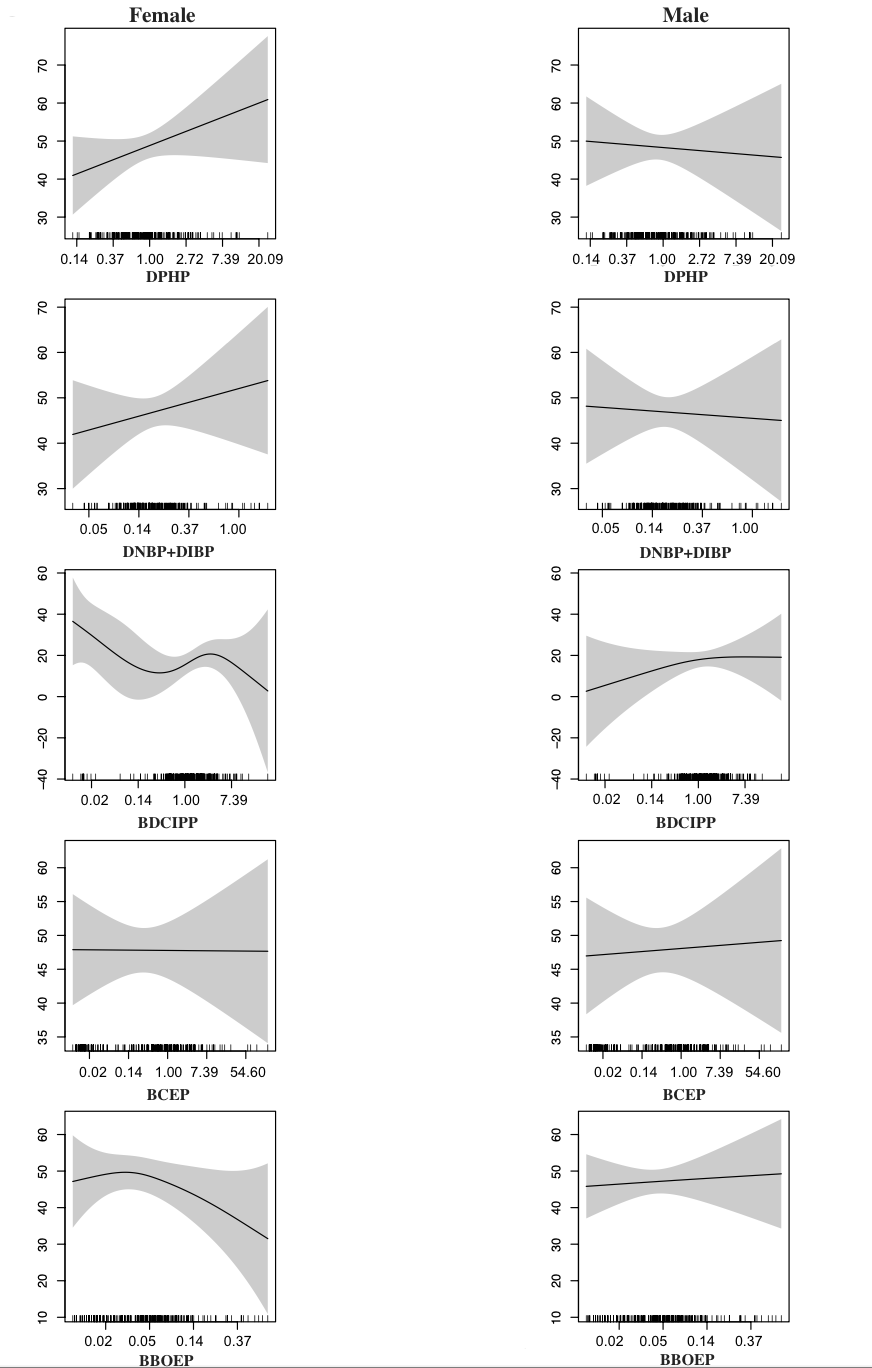 |
| All models adjusted for recruitment site, maternal age, race/ethnicity, household annual income, education, pre-pregnancy BMI, GA at sample collection, child adjusted age at CBCL administration, season, infant birth order, child sex. Note: OPE, Organophosphate Ester; CBCL, Child Behavior Checklist; DPHP, Diphenyl phosphate; DNBP+DIBP, Sum of Di-n-butyl phosphate and Di-isobutyl phosphate; BDCIPP, Bis(1,3-dichloro-2-propyl) phosphate; BCEP, Bis(2-chloroethyl) phosphate; BBOEP, Bis(butoxethyl) phosphate. |

| Supplemental Figure 5: Associations Between Urinary Prenatal OPE Metabolite Concentrations (ng/mL) and CBCL Composite T-Scores, Using Generalized Additive Models (N=204) |
| --- |
| 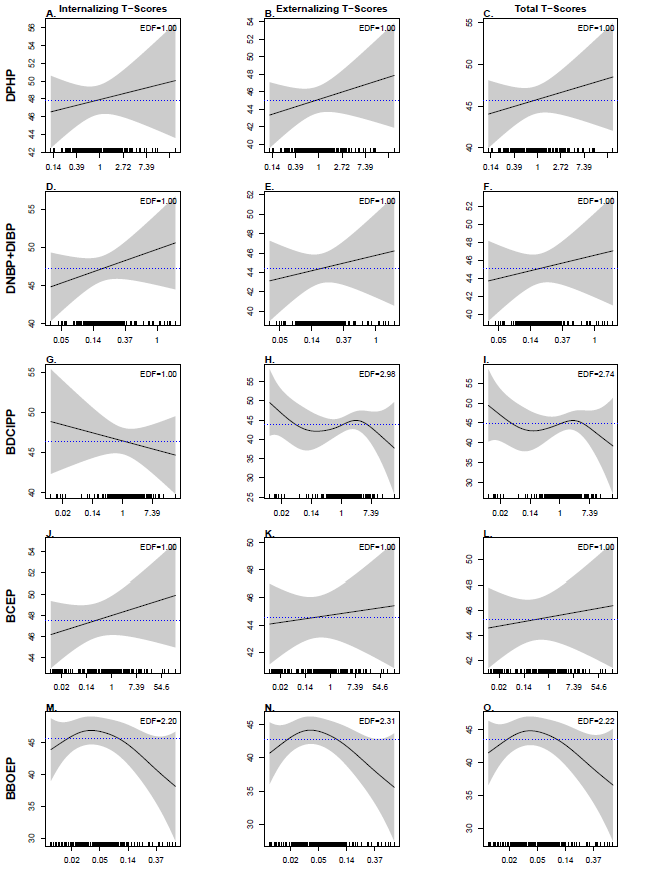  †  †  † |
| All models adjusted for recruitment site, maternal age, race/ethnicity, household annual income, education, pre-pregnancy BMI, GA at sample collection, child adjusted age at CBCL administration, season, infant birth order, child sex. Note: OPE, Organophosphate Ester; CBCL, Child Behavior Checklist; DPHP, Diphenyl phosphate; DNBP+DIBP, Sum of Di-n-butyl phosphate and Di-isobutyl phosphate; BDCIPP, Bis(1,3-dichloro-2-propyl) phosphate; BCEP, Bis(2-chloroethyl) phosphate; BBOEP, Bis(butoxethyl) phosphate.  †Significant non-linearity |

| Supplemental Figure 6: Associations Between Urinary Prenatal OPE Metabolite Concentrations (ng/mL) and CBCL Composite Raw Scores Among Participants Who Reported No In-Utero Smoking, Using Generalized Additive Models (N=199) |
| --- |
| 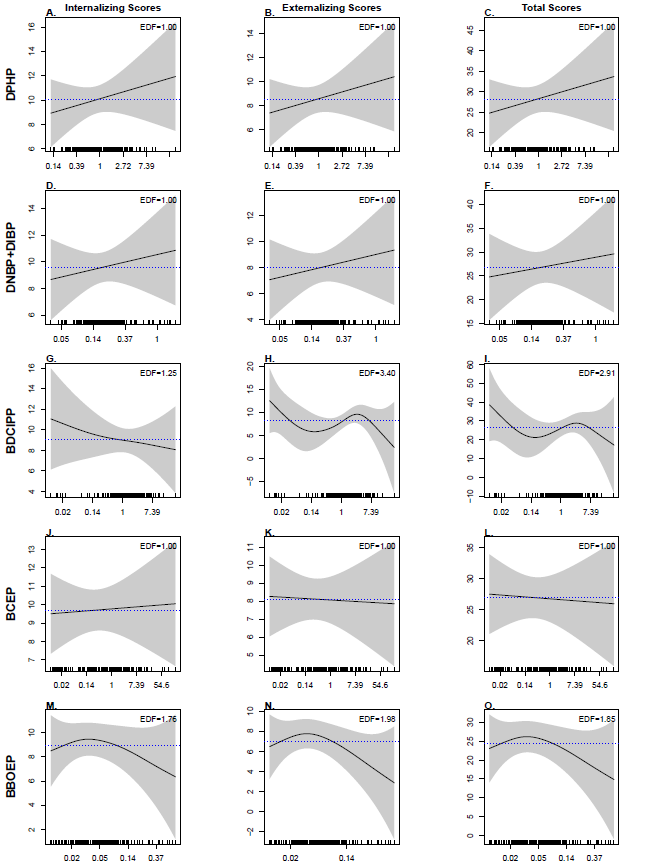  † |
| All models adjusted for recruitment site, maternal age, race/ethnicity, household annual income, education, pre-pregnancy BMI, GA at sample collection, child adjusted age at CBCL administration, season, infant birth order, child sex. Note: OPE, Organophosphate Ester; CBCL, Child Behavior Checklist; DPHP, Diphenyl phosphate; DNBP+DIBP, Sum of Di-n-butyl phosphate and Di-isobutyl phosphate; BDCIPP, Bis(1,3-dichloro-2-propyl) phosphate; BCEP, Bis(2-chloroethyl) phosphate; BBOEP, Bis(butoxethyl) phosphate.  †Significant non-linearity |

| Supplemental Figure 7: Posterior Inclusion Probabilities (PIPs) for Pairwise Interactions Between OPE Metabolites and CBCL Composite Raw Scores Using NLinteraction Method | | |
| --- | --- | --- |
| Internalizing | Externalizing | Total Problems |
| 1. 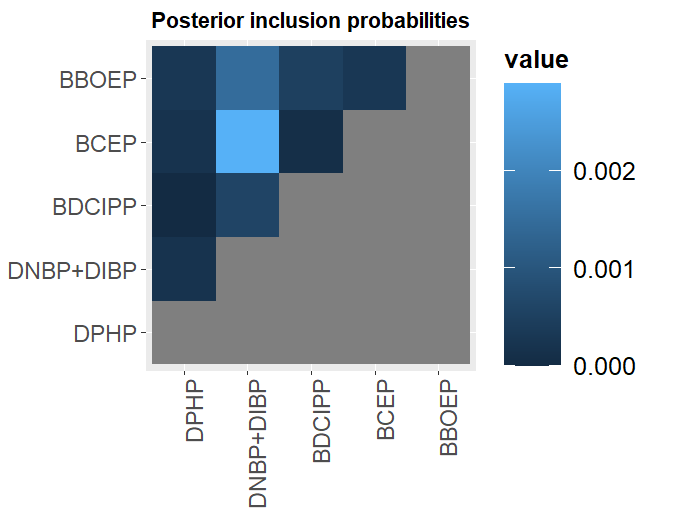 | 1. 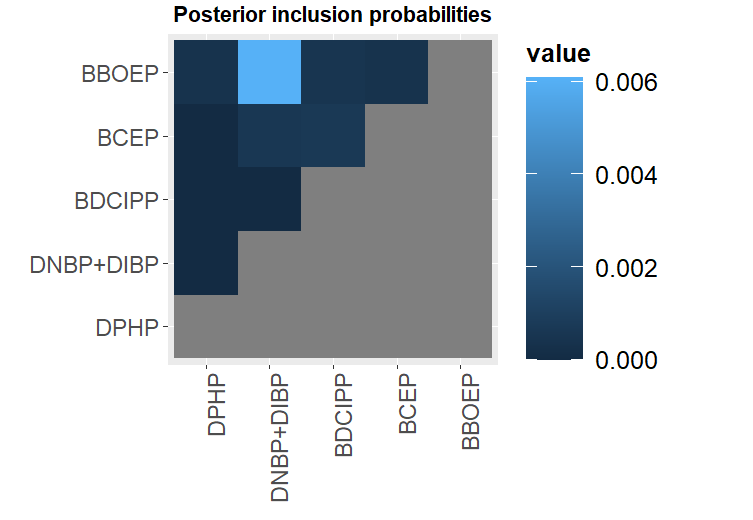 | 1. 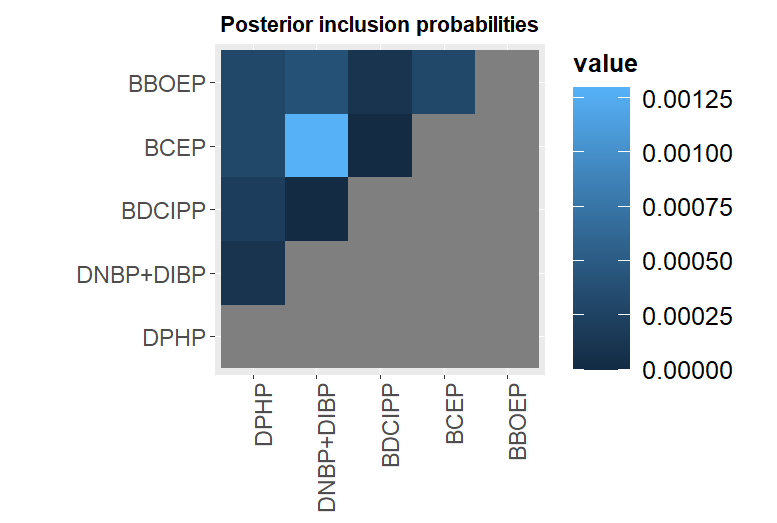 |
| Posterior inclusion probabilities for each of the five metabolites were estimated using the NLinteraction method. The light blue color reflects higher PIPs scores across metabolites. All models were adjusted for recruitment site, maternal age, race/ethnicity, household annual income, education, pre-pregnancy BMI, GA at sample collection, child adjusted age at CBCL administration, season, infant birth order, child sex. OPE metabolites and CBCL raw composite scores were natural log-transformed, mean centered, and standard deviation scaled. Continuous covariates were mean-centered and standard deviation scaled. Note: BKMR, Bayesian Kernel Machine Regression; OPE, Organophosphate Ester; CBCL, Child Behavior Checklist; DPHP, Diphenyl phosphate; DNBP+DIBP, Sum of Di-n-butyl phosphate and Di-isobutyl phosphate; BDCIPP, Bis(1,3-dichloro-2-propyl) phosphate; BCEP, Bis(2-chloroethyl) phosphate; BBOEP, Bis(butoxethyl) phosphate. | | |

| Supplemental Figure 8: Prenatal DNBP+DIBP Exposures and Children’s Total Problems Scores by Tertiles of BCEP, Using Generalized Additive Models |  |
| --- | --- |
| 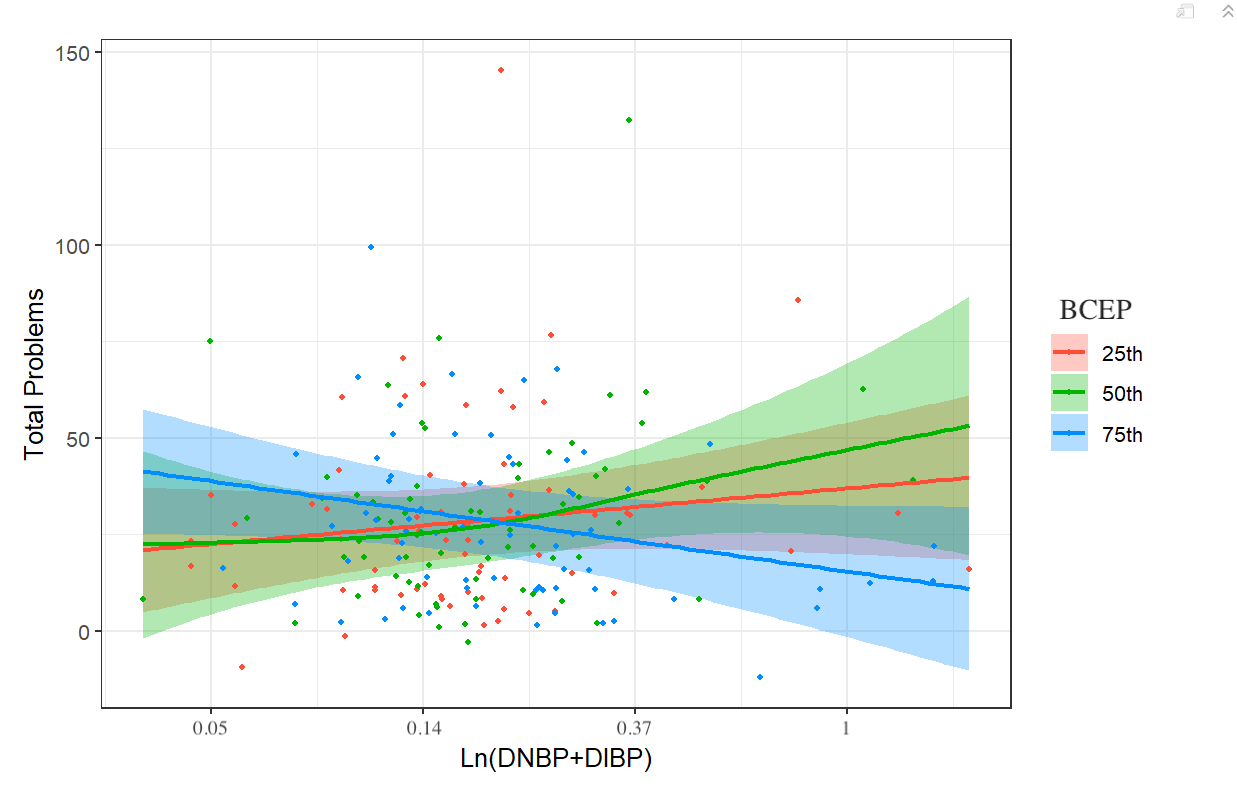 | |
| Note: DNBP+DIBP, Sum of Di-n-butyl phosphate and Di-isobutyl phosphate; BCEP, Bis(2-chloroethyl) phosphate. | |

| Supplemental Figure 9: Prenatal OPE Urinary Metabolite Mixtures (ng/mL) and CBCL Composite T-Scores, Using BKMR (N=204) | | | |
| --- | --- | --- | --- |
| Cumulative Mixture | Univariate Plots | Bivariate Plots | |
| Internalizing | | | |
| 1. 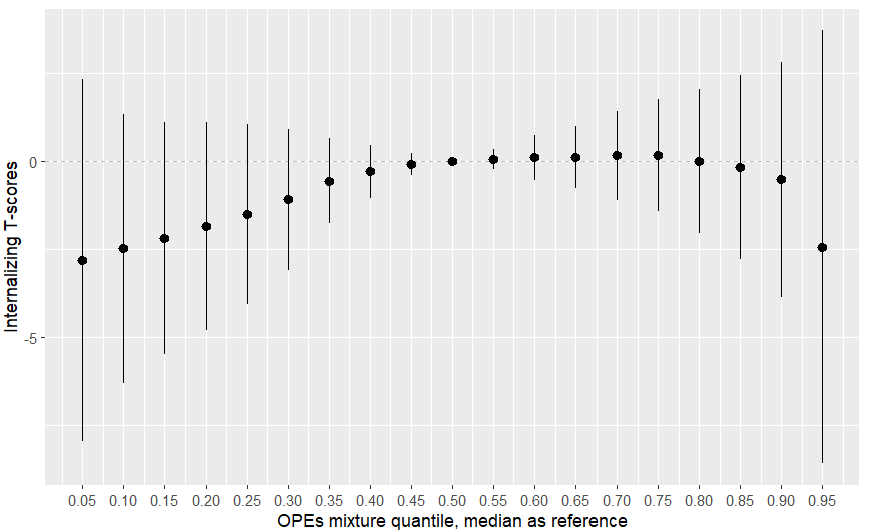 | 1. 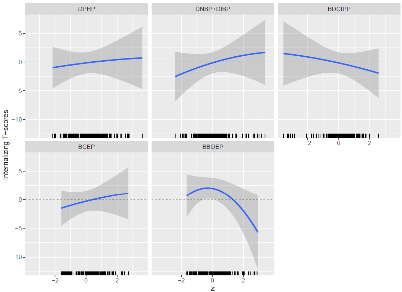 | 1. 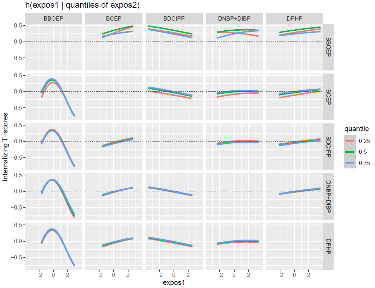 | |
| Externalizing | | | |
| 1. 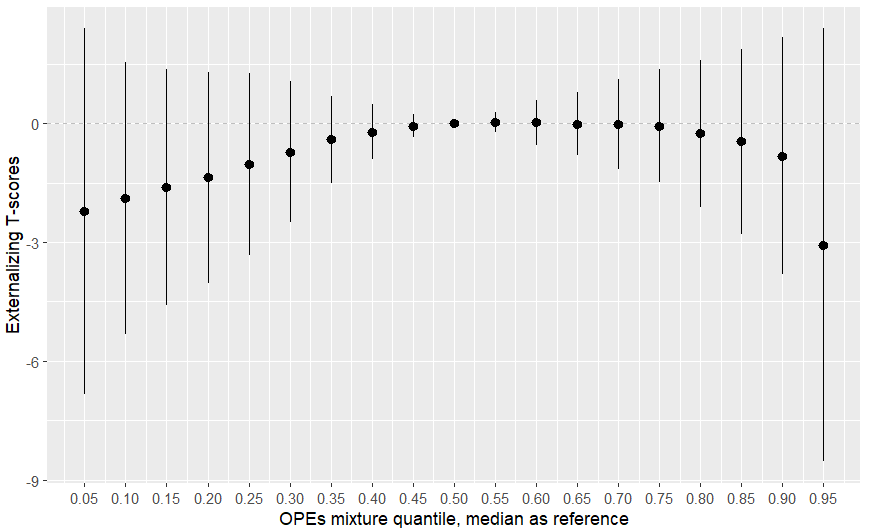 | 1. 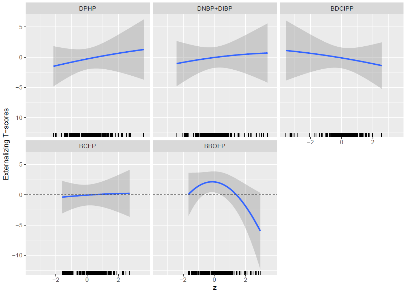 | 1. 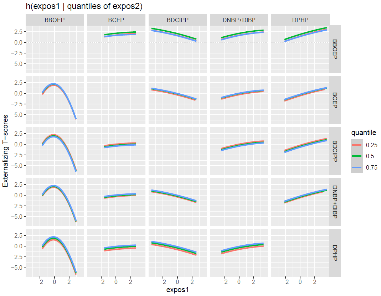 | |
| Total Problems | | | |
| 1. 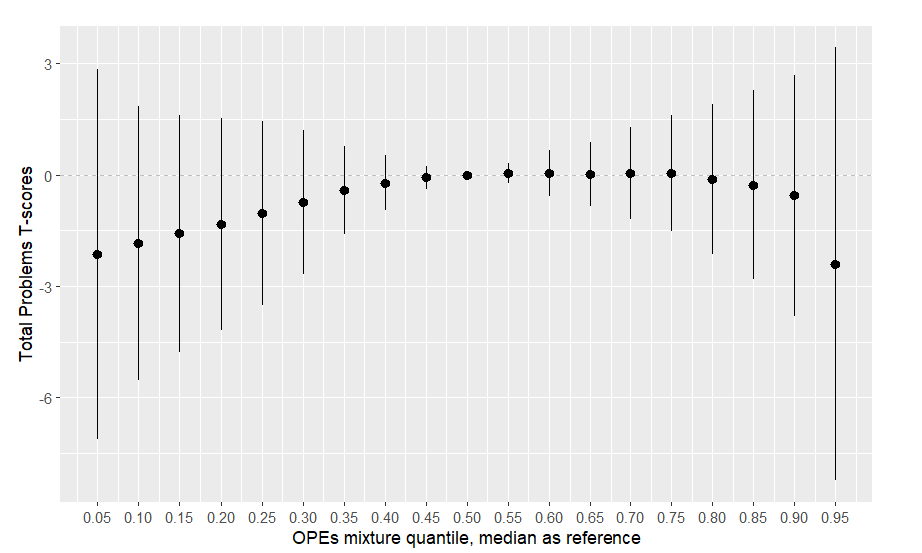 | 1. 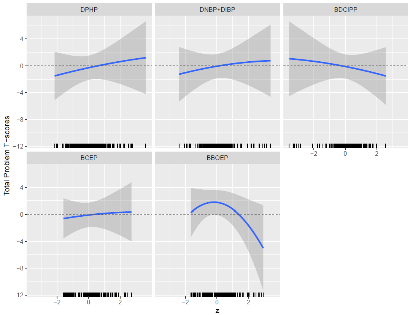 | 1. 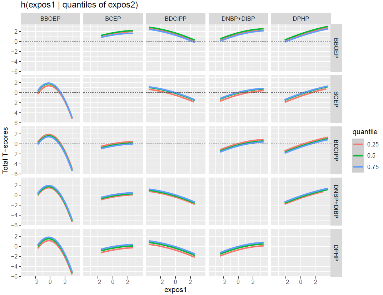 | |
| Figure 9 includes: 1) the estimated difference in CBCL composite score when setting all metabolites to the percentile specified on the x-axis compared with setting all metabolites to their median values (column 1), 2) the univariate relationship between each metabolite and CBCL outcome, while other metabolites are fixed at their medians, and a rug plot showing the distribution of the specified metabolite along the x-axis of each panel (column 2), and 3) the bivariate association between each OPE metabolite (labelled in the column) and CBCL composite score (Y axis), while setting a second metabolite (labelled in the row) to its 25^th^, 50^th^, and 75^th^ percentile and all other metabolites to their median. All models were adjusted for recruitment site, maternal age, race/ethnicity, household annual income, education, pre-pregnancy BMI, GA at sample collection, child adjusted age at CBCL administration, season, infant birth order, child sex. OPE metabolites were natural log-transformed, mean centered, and standard deviation scaled. Continuous covariates were mean-centered and standard deviation scaled. Note: BKMR, Bayesian Kernel Machine Regression; OPE, Organophosphate Ester; CBCL, Child Behavior Checklist; DPHP, Diphenyl phosphate; DNBP+DIBP, Sum of Di-n-butyl phosphate and Di-isobutyl phosphate; BDCIPP, Bis(1,3-dichloro-2-propyl) phosphate; BCEP, Bis(2-chloroethyl) phosphate; BBOEP, Bis(butoxethyl) phosphate. | | |  |

| Supplemental Figure 10: Prenatal OPE Urinary Metabolite Mixtures (ng/mL) and CBCL Composite Raw Scores Among Participants Who Reported No In-Utero Smoking, Using BKMR (N=199) | | | |
| --- | --- | --- | --- |
| Cumulative Mixture | Univariate Plots | Bivariate Plots | |
| Internalizing | | | |
| 1. 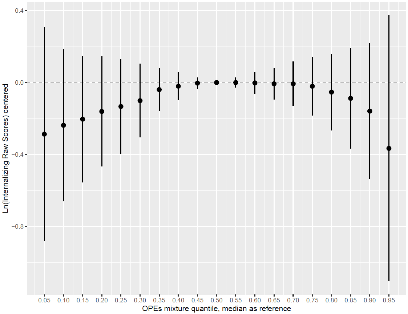 | 1. 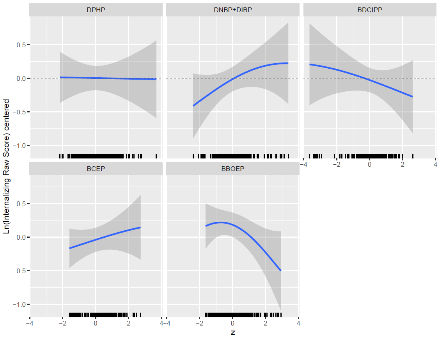 | 1. 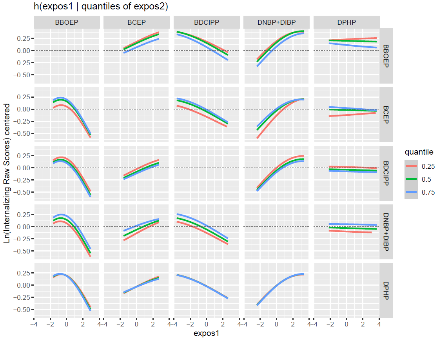 | |
| Externalizing | | | |
| 1. 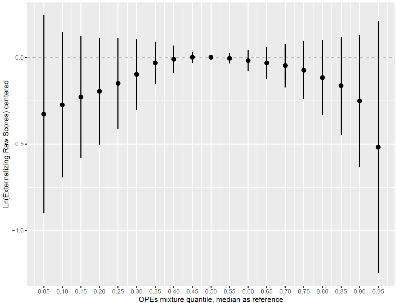 | 1. 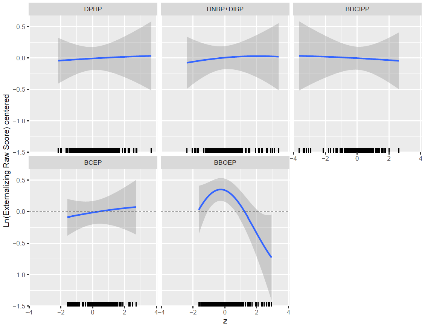 | 1. 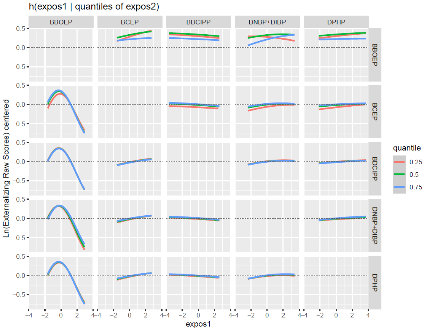 | |
| Total Problems | | | |
| 1. 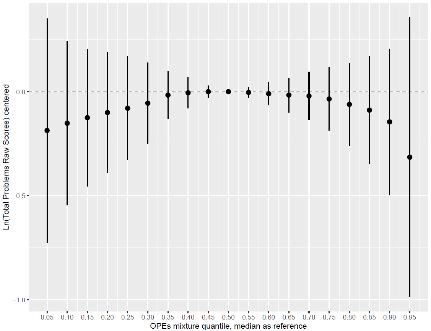 | 1. 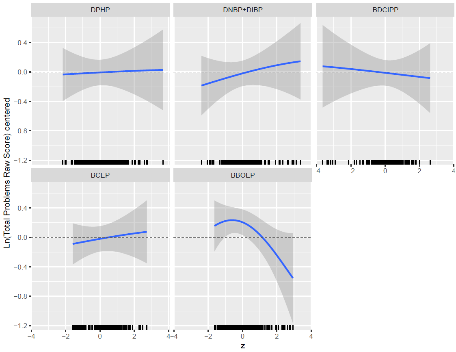 | 1. 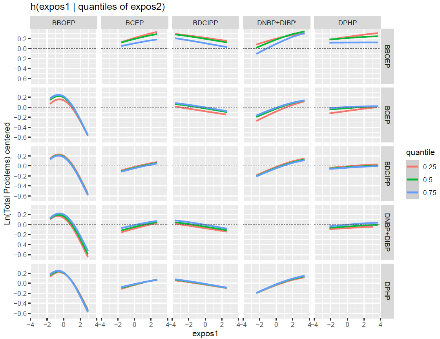 | |
| Figure 10 includes: 1) the estimated difference in CBCL composite score when setting all metabolites to the percentile specified on the x-axis compared with setting all metabolites to their median values (column 1), 2) the univariate relationship between each metabolite and CBCL outcome, while other metabolites are fixed at their medians, and a rug plot showing the distribution of the specified metabolite along the x-axis of each panel (column 2), and 3) the bivariate association between each OPE metabolite (labelled in the column) and CBCL composite score (Y axis), while setting a second metabolite (labelled in the row) to its 25^th^, 50^th^, and 75^th^ percentile and all other metabolites to their median (column 3). All models were adjusted for recruitment site, maternal age, race/ethnicity, household annual income, education, pre-pregnancy BMI, GA at sample collection, child adjusted age at CBCL administration, season, infant birth order, child sex. OPE metabolites and CBCL raw composite scores were natural log-transformed, mean centered, and standard deviation scaled. Continuous covariates were mean-centered and standard deviation scaled. Note: BKMR, Bayesian Kernel Machine Regression; OPE, Organophosphate Ester; CBCL, Child Behavior Checklist; DPHP, Diphenyl phosphate; DNBP+DIBP, Sum of Di-n-butyl phosphate and Di-isobutyl phosphate; BDCIPP, Bis(1,3-dichloro-2-propyl) phosphate; BCEP, Bis(2-chloroethyl) phosphate; BBOEP, Bis(butoxethyl) phosphate. | | |  |

| Supplemental Figure 11: Prenatal OPE Urinary Metabolite Mixtures (ng/mL) and CBCL Composite Raw Scores, Using BKMR Varying the Smoothing Parameter to b=50 | | | |
| --- | --- | --- | --- |
| Cumulative Mixture | Univariate Plots | Bivariate Plots | |
| Internalizing | | | |
| 1. 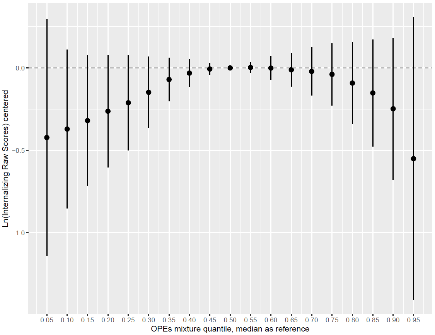 | 1. 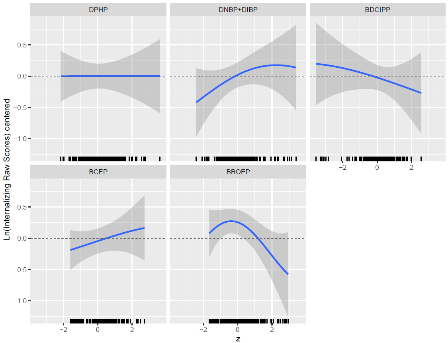 | 1. 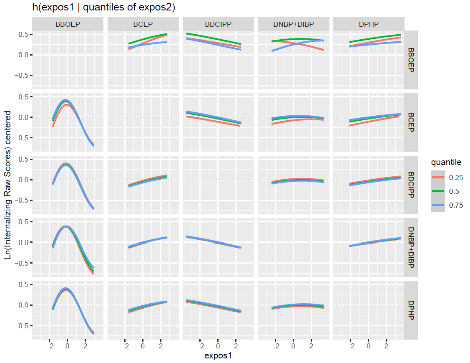 | |
| Externalizing | | | |
| 1. 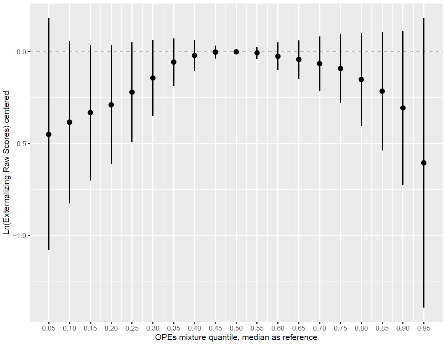 | 1. 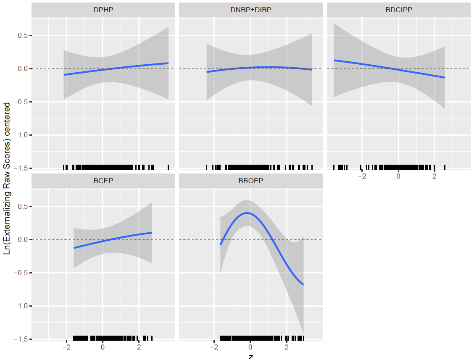 | 1. 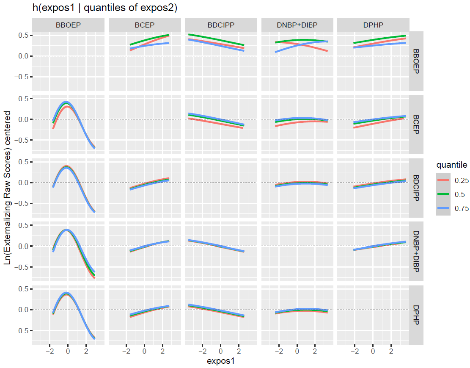 | |
| Total Problems | | | |
| 1. 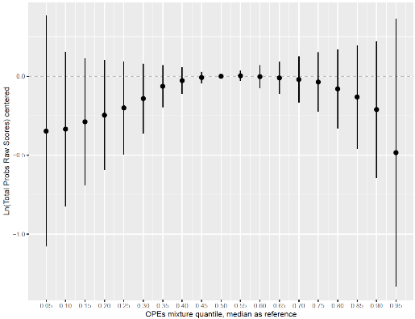 | 1. 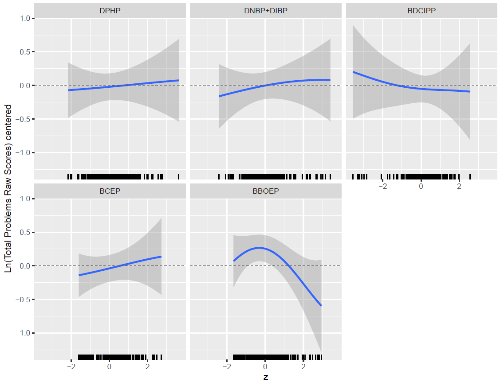 | 1. 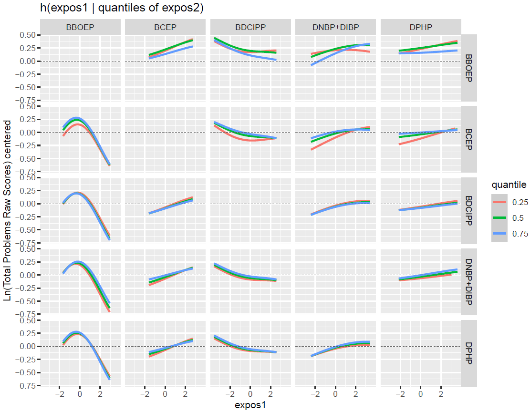 | |
| Figure 11 includes: 1) the estimated difference in CBCL composite score when setting all metabolites to the percentile specified on the x-axis compared with setting all metabolites to their median values (column 1), 2) the univariate relationship between each metabolite and CBCL outcome, while other metabolites are fixed at their medians, and a rug plot showing the distribution of the specified metabolite along the x-axis of each panel (column 2), and 3) the bivariate association between each OPE metabolite (labelled in the column) and CBCL composite score (Y axis), while setting a second metabolite (labelled in the row) to its 25^th^, 50^th^, and 75^th^ percentile and all other metabolites to their median. All models were adjusted for recruitment site, maternal age, race/ethnicity, household annual income, education, pre-pregnancy BMI, GA at sample collection, child adjusted age at CBCL administration, season, infant birth order, child sex. OPE metabolites and CBCL raw composite scores were natural log-transformed, mean centered, and standard deviation scaled. Continuous covariates were mean-centered and standard deviation scaled. Note: BKMR, Bayesian Kernel Machine Regression; OPE, Organophosphate Ester; CBCL, Child Behavior Checklist; DPHP, Diphenyl phosphate; DNBP+DIBP, Sum of Di-n-butyl phosphate and Di-isobutyl phosphate; BDCIPP, Bis(1,3-dichloro-2-propyl) phosphate; BCEP, Bis(2-chloroethyl) phosphate; BBOEP, Bis(butoxethyl) phosphate. | | |  |

| Supplemental Figure 12: Prenatal OPE Urinary Metabolite Mixtures (ng/mL) and CBCL Composite Raw Scores, Using BKMR Varying the Smoothing Parameter to b=1000 | | | |
| --- | --- | --- | --- |
| Cumulative Mixture | Univariate Plots | Bivariate Plots | |
| Internalizing | | | |
| 1. 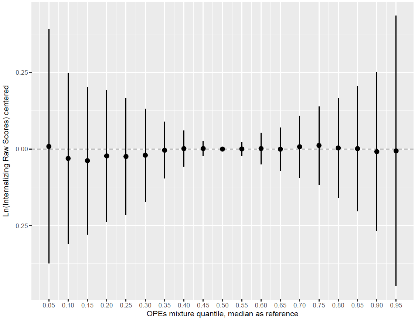 | 1. 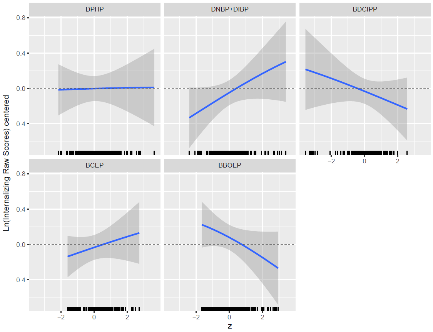 | 1. 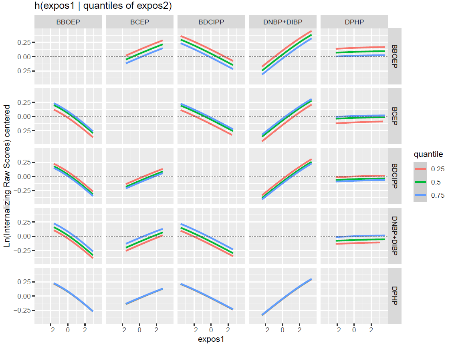 | |
| Externalizing | | | |
| 1. 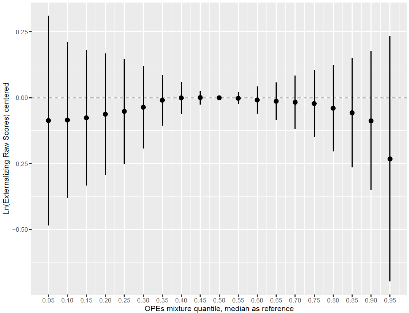 | 1. 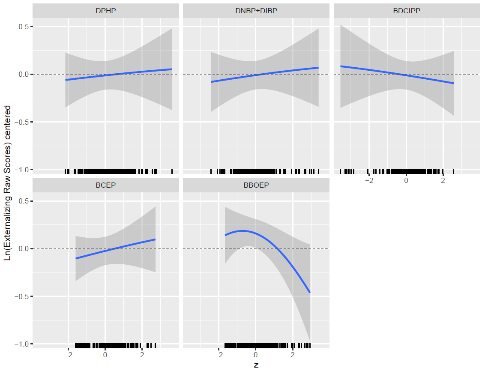 | 1. 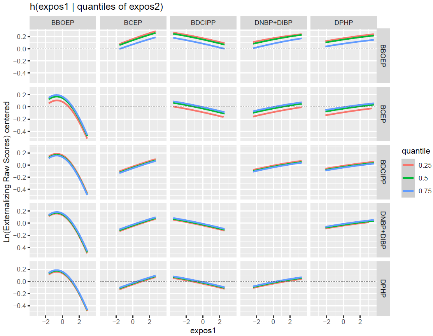 | |
| Total Problems | | | |
| 1. 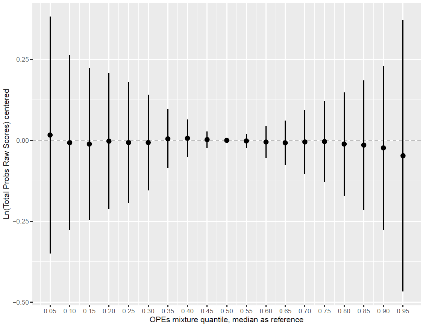 | 1. 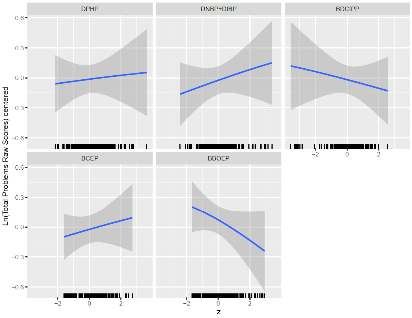 | 1. 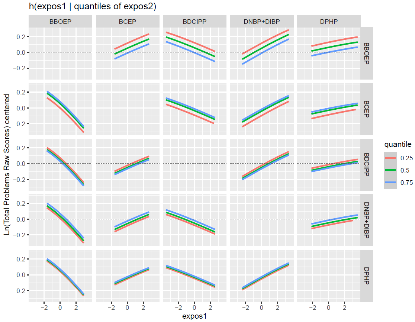 | |
| Figure 12 includes: 1) the estimated difference in CBCL composite score when setting all metabolites to the percentile specified on the x-axis compared with setting all metabolites to their median values (column 1), 2) the univariate relationship between each metabolite and CBCL outcome, while other metabolites are fixed at their medians, and a rug plot showing the distribution of the specified metabolite along the x-axis of each panel (column 2), and 3) the bivariate association between each OPE metabolite (labelled in the column) and CBCL composite score (Y axis), while setting a second metabolite (labelled in the row) to its 25^th^, 50^th^, and 75^th^ percentile and all other metabolites to their median. All models were adjusted for recruitment site, maternal age, race/ethnicity, household annual income, education, pre-pregnancy BMI, GA at sample collection, child adjusted age at CBCL administration, season, infant birth order, child sex. OPE metabolites and CBCL raw composite scores were natural log-transformed, mean centered, and standard deviation scaled. Continuous covariates were mean-centered and standard deviation scaled. Note: BKMR, Bayesian Kernel Machine Regression; OPE, Organophosphate Ester; CBCL, Child Behavior Checklist; DPHP, Diphenyl phosphate; DNBP+DIBP, Sum of Di-n-butyl phosphate and Di-isobutyl phosphate; BDCIPP, Bis(1,3-dichloro-2-propyl) phosphate; BCEP, Bis(2-chloroethyl) phosphate; BBOEP, Bis(butoxethyl) phosphate. | | |  |

| Supplemental Figure 13: Posterior Inclusion Probabilities (PIPs) for Pairwise Interactions Between OPE Metabolites and CBCL Composite Raw Scores Using NLinteraction Method and Increasing the Threshold to 0.25 | | |
| --- | --- | --- |
| Internalizing | Externalizing | Total Problems |
| 1. 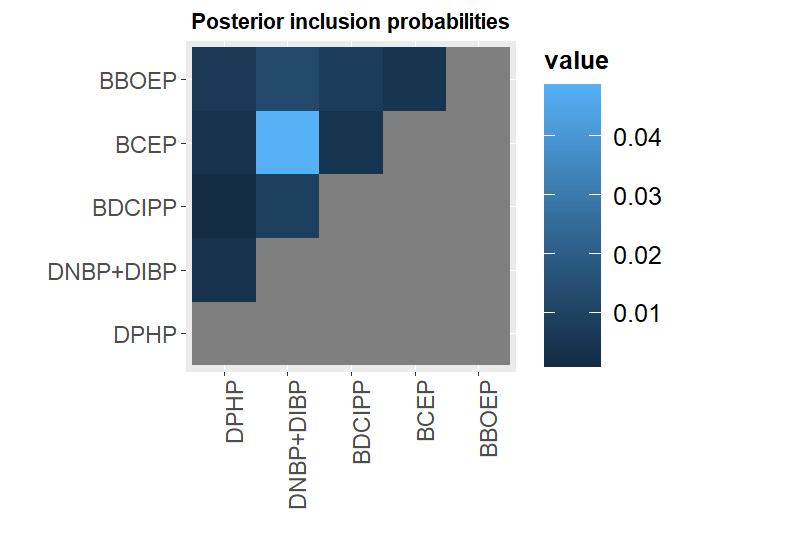 | 1. 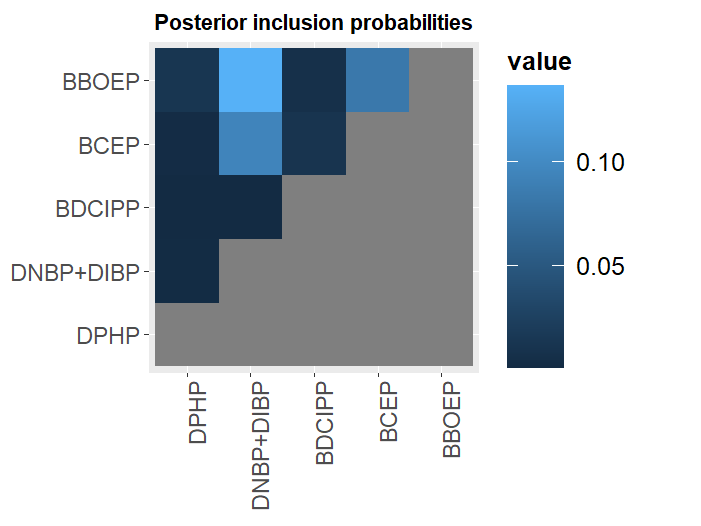 | 1. 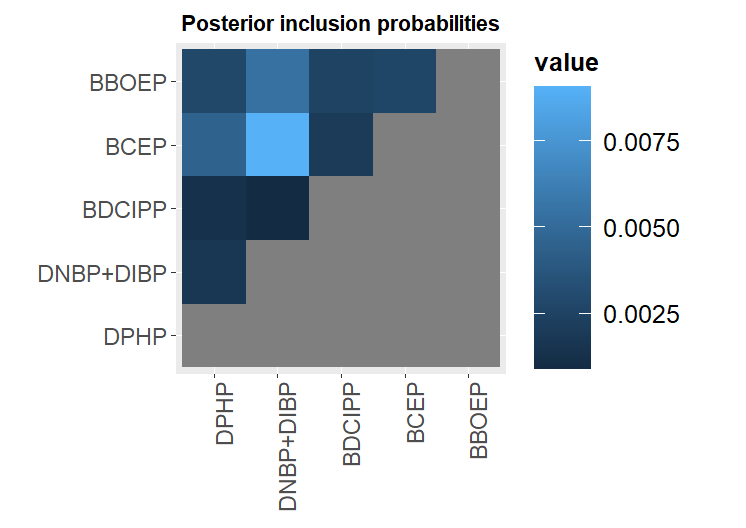 |
| Posterior inclusion probabilities for each of the five metabolites were estimated using the NLinteraction method. The light blue color reflects higher PIPs scores across metabolites. All models were adjusted for recruitment site, maternal age, race/ethnicity, household annual income, education, pre-pregnancy BMI, GA at sample collection, child adjusted age at CBCL administration, season, infant birth order, child sex. OPE metabolites and CBCL raw composite scores were natural log-transformed, mean centered, and standard deviation scaled. Continuous covariates were mean-centered and standard deviation scaled. Note: BKMR, Bayesian Kernel Machine Regression; OPE, Organophosphate Ester; CBCL, Child Behavior Checklist; DPHP, Diphenyl phosphate; DNBP+DIBP, Sum of Di-n-butyl phosphate and Di-isobutyl phosphate; BDCIPP, Bis(1,3-dichloro-2-propyl) phosphate; BCEP, Bis (2- chloroethyl) phosphate; BBOEP, Bis(butoxethyl) phosphate. | | |

| Supplemental Figure 14: Prenatal OPE Urinary Metabolite Mixtures and CBCL Composite Raw Scores, Using BKMR and Metabolites with Detect Frequency >80% Only (N=204) | |
| --- | --- |
|  |  |
| Figure 14 includes: 1) the estimated difference in CBCL composite score when setting all metabolites to the percentile specified on the x-axis compared with setting all metabolites to their median values (column 1), 2) the univariate relationship between each metabolite and CBCL outcome, while other metabolites are fixed at their medians (column 2). All models were adjusted for recruitment site, maternal age, race/ethnicity, household annual income, education, pre-pregnancy BMI, GA at sample collection, child adjusted age at CBCL administration, season, infant birth order, child sex. OPE metabolites and CBCL raw scores were natural log-transformed, mean centered, and standard deviation scaled. Continuous covariates were mean-centered and standard deviation scaled. Note: BKMR, Bayesian Kernel Machine Regression; OPE, Organophosphate Ester; CBCL, Child Behavior Checklist; DPHP, Diphenyl phosphate; DNBP+DIBP, Sum of Di-n-butyl phosphate and Di-isobutyl phosphate; BDCIPP. | |

| Supplemental Figure 15: Bivariate Associations Between Prenatal OPE Urinary Metabolite Mixtures (ng/mL) and CBCL Composite Raw Scores, Using BKMR and Metabolites with Detect Frequency >80% Only (N=204) |
| --- |
| Bivariate Plots |
|  |
| Figure 15 shows the bivariate association between each OPE metabolite (labelled in the column) and CBCL composite score (Y axis), while setting a second metabolite (labelled in the row) to its 25^th^, 50^th^, and 75^th^ percentile and all other metabolites to their median. All models were adjusted for recruitment site, maternal age, race/ethnicity, household annual income, education, pre-pregnancy BMI, GA at sample collection, child adjusted age at CBCL administration, season, infant birth order, child sex. OPE metabolites and CBCL raw scores were natural log-transformed, mean centered, and standard deviation scaled. Continuous covariates were mean-centered and standard deviation scaled. Note: BKMR, Bayesian Kernel Machine Regression; OPE, Organophosphate Ester; CBCL, Child Behavior Checklist; DPHP, Diphenyl phosphate; DNBP+DIBP, Sum of Di-n-butyl phosphate and Di-isobutyl phosphate; BDCIPP, Bis(1,3-dichloro-2-propyl) phosphate. |

| Supplemental Table 1: Parent Compounds of OPE Metabolites Analyzed and Common Applications | | | |
| --- | --- | --- | --- |
| Metabolite (Abbr) | Parent Compound (Abbr) | Chemical Group | Common Applications |
| Diphenyl phosphate (DPHP) | Triphenyl phosphate (TPHP) | Aryl | Foam seating and bedding, plastic and rubber products, and nail polish (industrial; commercial; consumer). FDA indirect additives used in food contact substances. |
| Di-n-butyl phosphate (DNBP) + Di-isobutyl phosphate (DIBP) | Tri-n-butyl Phosphate (TNBP)  Tris(isobutyl) phosphate (TIBP) | Alkyl  Alkyl | Adhesives/sealants and inks/toners. (industrial; commercial; consumer) FDA indirect additives used in food contact substances. |
| Bis(1,3-dichloro-2-propyl) phosphate (BDCIPP) | Tris(1,3-dichloro-2-propyl)phosphate (TDCIPP) | Chlorinated Alkyl | Polyurethane foam seating and bedding (industrial; commercial; consumer). |
| Bis(2-chloroethyl) phosphate (BCEP) | Tri(2-chloroethyl)phosphate (TCEP) | Chlorinated Alkyl | Industrial, unspecified. |
| Bis(butoxethyl) phosphate (BBOEP) | Tris(2-butoxyethyl) phosphate (TBOEP) | Alkyl | Plasticizers for most resins and elastomers; floor finishes and waxes; flame-retarding agent; adhesives and seal (industrial, commercial, consumer). |
| Bis(1-chloro-2-propyl) phosphate (BCIPP) | Tris(1-chloro-2-propyl)phosphate (TCIPP) | Chlorinated Alkyl | Foam insulation, building/construction materials, foam seating and bedding products, and electronic products (industrial; commercial; consumer). |
| Bis(2-methylphenyl) phosphate (BMPP) | Trimethylphenyl phosphate (TMPP) (formerly Tricresyl phosphate (TCP)) | Aryl | Plastic and rubber product, lubricants and greases, and other unspecified uses. (industrial; commercial; consumer). |
| Bis(2-ethylhexyl) phosphate (BEHP) | Tris(2-ethylhexyl) phosphate (TEHP) | Alkyl | Lubricants and additives, oxidizing/reducing agents, processing aids, agricultural pesticides, plastic and rubber products. |
| Dipropyl phosphate (DPRP) | Tripropyl phosphate (TPP) | Aryl | Plasticizer. |
| Note: Abbr, abbreviation; OPE, Organophosphate Ester  *Data compiled using Pub Chem and Hou et al. (2016) | | | |

| Supplemental Table 2. Distribution of Specific Gravity Adjusted OPE Concentrations (ng/mL) in Urine for Maternal Participants Analyzed (N=204) vs the Full Sample of Maternal Participants with OPEs Available (N=426) | | | | | | | | | | | |
| --- | --- | --- | --- | --- | --- | --- | --- | --- | --- | --- | --- |
| Subset Analyzed (N=204) | | | | | | Available OPEs (N=426) | | | | | |
| Metabolite | 25th | 50th | 75th | Min-Max | Detect  Frequency | 25th | 50th | 75th | Min-Max | Detect Frequency | LOD (ng/mL) |
| DPHP | 0.47 | 0.83 | 1.47 | 0.12-25.59 | 99.51% | 0.47 | 0.77 | 1.46 | 0.12-25.59 | 99.77% | 0.0281 |
| DNBP+DIBP | 0.12 | 0.17 | 0.25 | ND-1.78 | 96.57% | 0.12 | 0.18 | 0.26 | ND-4.29 | 97.65% | 0.0441 |
| BDCIPP | 0.61 | 1.26 | 2.14 | ND-34.94 | 95.10% | 0.61 | 1.29 | 2.29 | ND-68.00 | 94.60% | 0.0174 |
| BCEP | 0.02 | 0.47 | 1.60 | ND-168.00 | 68.63% | 0.02 | 0.53 | 1.62 | ND-168.00 | 68.31% | 0.0200 |
| BBOEP | 0.02 | 0.04 | 0.08 | ND-0.74 | 63.24% | 0.02 | 0.04 | 0.07 | ND-1.17 | 64.79% | 0.0199 |
| BCIPP | ND | 0.12 | 0.71 | ND-19.90 | 51.47% | ND | 0.18 | 0.77 | ND-40.56 | 53.76% | 0.0204 |
| BMPP | ND | 0.01 | 0.04 | ND-0.47 | 39.71% | ND | 0.01 | 0.04 | ND-0.69 | 38.73% | 0.0115 |
| BEHP | ND | ND | 0.03 | ND-3.48 | 25.00% | ND | ND | 0.04 | ND-4.42 | 25.59% | 0.0170 |
| DPRP | ND | ND | 0.06 | ND-2.85 | 25.98% | ND | ND | 0.05 | ND-2.85 | 24.18% | 0.0278 |
| Note: OPE, Organophosphate Esters; LOD, Limit of Detection; ND, Non-detect; DPHP, Diphenyl phosphate; DNBP+DIBP, Sum of Di-n-butyl phosphate and Di-isobutyl phosphate; BDCIPP, Bis(1,3-dichloro-2-propyl) phosphate; BCEP, Bis(2-chloroethyl) phosphate; BBOEP, Bis(butoxethyl) phosphate; BCIPP, Bis(1-chloro-2-propyl) phosphate; BMPP, Bis(2-methylphenyl) phosphate; BEHP, Bis(2-ethylhexyl) phosphate; DPRP, Dipropyl phosphate; Min, Minimum; Max, Maximum. | | | | | | | | | | | |

| Supplemental Table 3: Comparison of Participant Characteristics Analyzed in the Analytical Dataset (N=204) to Subset with OPE Metabolite Concentrations Available (N=426) and Full MADRES Participants Who Delivered Children in the Study by August 28^th^, 2022 (N=774) | | | |  |
| --- | --- | --- | --- | --- |
|  | Analytical Subset  Mean (SD)/Freq(%) | Available Metabolites  Mean (SD)/Freq(%) | Full MADRES Cohort  Mean (SD)/Freq(%) |  |
| **Maternal Characteristics** | N=204 | N=426 | N=774 |  |
| Age (years) | 29.4 (5.9) | 28.9 (6.1) | 28.3 (6.0) |  |
| Education       ≤High School       ≥High School  Missing | 113 (55.4%)  91 (44.6%)  ─ | 240 (56.3%)  182 (42.7%)  4 (0.9%) | 416 (53.7%)  316 (40.8%)  42 (5.4%) |  |
| Income       Don’t Know       Less than $50,000       ≥ $50,000  Missing | 55 (27.0%)  118 (57.8%)  31 (15.2%)  ─ | 122 (28.6%)  249 (58.5%)  51 (12.0%)  4 (0.9%) | 244 (31.5%)  416 (53.7%)  73 (9.4%)  41 (5.3%) |  |
| NIH Race Categories       White, non-Hispanic       Black, non-Hispanic       Hispanic       Multiracial/other, non-Hispanic  Missing | 17 (8.3%)  21 (10.3%)  161 (78.9%)  5 (2.5%)  ─ | 29 (6.8%)  49 (11.5%)  329 (77.2%)  15 (3.5%)  4 (0.9%) | 40 (5.2%)  87 (11.2%)  579 (74.8%)  26 (3.4%)  42 (5.4%) |  |
| Smoking During Pregnancy       No       Yes  Missing | 199 (98.0%)  5 (2.5%)  ─ | 418 (98.1%)  8 (1.9%)  ─ | 659 (85.1%)  13 (1.7%)  102 (13.2%) |  |
| Pre-pregnancy BMI (kg/m^2^) | 29.1 (6.5) | 28.6 (6.7) | 28.7 (6.7) |  |
| **Infant Characteristics** |  |  |  |  |
| Sex       Female       Male  Missing | 105 (51.5%)  99 (48.5%)  ─ | 218 (51.2%)  208 (48.8)  ─ | 384 (49.6%)  387 (50.0%)  3 (0.4%) |  |
| Infant Birth Order       First Born       Second or more       Missing | 74 (36.3%)  123 (60.3%)  7 (3.4%) | 147 (34.5%)  261 (61.3%)  18 (4.2) | 242 (31.3%)  406 (52.5%)  126 (16.3%) |  |
| Gestational Age at Birth (weeks)  Child Adjusted Age at CBCL Administration (weeks)^a^ | 39.1 (1.5)  155.8 (2.3) | 39.1 (1.5)  ─ | 39.0 (1.8)  ─ |  |

^a^Child age at questionnaire administration corrected for preterm birth (<37 weeks).

| Supplemental Table 4: Individual Associations Between Third Trimester Urinary OPE Metabolites (ng/mL) and CBCL Raw Composite Scores by Child Sex (N=204) | | | | | | | |
| --- | --- | --- | --- | --- | --- | --- | --- |
|  | Internalizing | | Externalizing | | Total Problems | | |
|  | Female Only  $\beta$ (95% CI)  (N=105) | Male Only  $\beta$ (95% CI)  (N=99) | Female Only  $\beta$ (95% CI)  (N=105) | Male Only  $\beta$ (95% CI)  (N=99) | | Female Only  $\beta$ (95% CI)  (N=105) | Male Only  $\beta$ (95% CI)  (N=99) |
| DPHP  T1 (<0.55)  T2 (0.55-1.15)  T3 (≥1.15) | \| REF  0.98 (0.49, 1.99)  1.01 (0.49, 2.09) \| \| --- \| | \| REF  0.82 (0.44, 1.55)  1.03 (0.55, 1.93) \| \| --- \| | \| REF  0.93 (0.53, 1.64)  1.11 (0.62, 1.98) \| \| --- \| | \| REF  0.87 (0.49, 1.53)  0.95 (0.54, 1.67) \| \| --- \| | | \| REF  0.92 (0.55, 1.55)  1.11 (0.65, 1.90) \| \| --- \| | \| REF  0.87 (0.57, 1.34)  0.97 (0.63, 1.48) \| \| --- \| |
| DNBP+DIBP  T1 (<0.14)  T2 (0.14-0.21)  T3 (≥0.21) | \| REF  0.93 (0.46, 1.91)  0.86 (0.43, 1.73) \| \| --- \| | \| REF  1.12 (0.60, 2.06)  1.14 (0.60, 2.15) \| \| --- \| | \| REF  0.85 (0.48, 1.51)  1.10 (0.63, 1.92) \| \| --- \| | \| REF  1.14 (0.66, 1.97)  0.92 (0.52, 1.62) \| \| --- \| | | \| REF  0.84 (0.50, 1.42)  0.92 (0.55, 1.54) \| \| --- \| | \| REF  1.07 (0.71, 1.62)  0.99 (0.64, 1.51) \| \| --- \| |
| BDCIPP  T1 (<0.85)  T2 (0.85-1.83)  T3 (≥1.83) | \| REF  0.65 (0.31, 1.36)  0.48 (0.22, 1.03) \| \| --- \| | \| 1.45 (0.77, 2.73)  1.30 (0.66, 2.56) \| \| --- \| | \| REF  0.71 (0.39, 1.29)  0.92 (0.49, 1.72) \| \| --- \| | \| REF  1.31 (0.74, 2.31)  1.19 (0.64, 2.18) \| \| --- \| | | \| REF  0.77 (0.45, 1.34)  0.86 (0.48, 1.52) \| \| --- \| | \| REF  1.21 (0.79, 1.85)  1.08 (0.68, 1.71) \| \| --- \| |
| BCEP  T1 (Non-detect)  T2 (0.04-0.97)  T3 (≥0.97) | \| REF  1.35 (0.67, 2.71)  1.08 (0.53, 2.20) \| \| --- \| | \| REF  1.19 (0.62, 2.28)  1.07 (0.55, 2.07) \| \| --- \| | \| REF  1.21 (0.69, 2.12)  1.13 (0.64, 2.00) \| \| --- \| | \| REF  1.17 (0.65, 2.08)  1.02 (0.56, 1.84) \| \| --- \| | | \| REF  1.13 (0.68, 1.90)  1.10 (0.65, 1.86) \| \| --- \| | \| REF  1.02 (0.66, 1.57)  0.99 (0.64, 1.55) \| \| --- \| |
| BBOEP  T1 (Non-detect)  T2 (0.01-0.06)  T3 (≥0.06) | \| REF  1.13 (0.58, 2.19)  0.53 (0.25, 1.12) \| \| --- \| | \| REF  1.13 (0.60, 2.12)  0.72 (0.37, 1.38) \| \| --- \| | \| REF  1.44 (0.85, 2.44)  0.69 (0.39, 1.25) \| \| --- \| | \| REF  1.28 (0.72, 2.26)  0.89 (0.49, 1.62) \| \| --- \| | | \| REF  1.19 (0.74, 1.92)  0.55 (0.32, 0.94)* \| \| --- \| | \| REF  1.11 (0.72, 1.71)  0.89 (0.57, 1.39) \| \| --- \| |
| BCIPP  T1 (Non-detect)  T2 (0.03- 0.66)  T3 (≥0.66) | \| REF  0.55 (0.29, 1.06)  0.61 (0.30, 1.25) \| \| --- \| | \| REF  1.32 (0.68, 2.55)  2.20 (1.23, 3.95)* \| \| --- \| | \| REF  0.75 (0.44, 1.27)  0.77 (0.43, 1.39) \| \| --- \| | \| REF  1.40 (0.77, 2.54)  1.68 (0.98, 2.86) \| \| --- \| | | \| REF  0.70 (0.43, 1.13)  0.69 (0.40, 1.17) \| \| --- \| | \| REF  1.35 (0.86, 2.10)  1.57 (1.06, 2.34)* \| \| --- \| |
| BMPP  Non-detect  Detect | REF  1.37 (0.75, 2.49) | REF   \| 1.11 (0.65, 1.88) \| \| --- \| | \| REF  1.69 (1.05, 2.70)* \| \| --- \| | \| REF  1.02 (0.64, 1.64) \| \| --- \| | | \| REF  1.51 (0.97, 2.33) \| \| --- \| | \| REF  0.94 (0.66, 1.35) \| \| --- \| |
| BEHP  Non-detect  Detect | REF   \| 1.19 (0.61, 2.32) \| \| --- \| | REF   \| 1.10 (0.62, 1.95) \| \| --- \| | \| REF  1.06 (0.62, 1.82) \| \| --- \| | \| REF  0.96 (0.58, 1.60) \| \| --- \| | | REF   \| 1.08 (0.66, 1.76) \| \| --- \| | \| REF  1.07 (0.73, 1.57) \| \| --- \| |
| DPRP  Non-detect  Detect | REF   \| 0.57 (0.30, 1.06) \| \| --- \| | REF  1.31 (0.72, 2.38) | REF   \| 0.73 (0.44, 1.21) \| \| --- \| | REF   \| 1.58 (0.93, 2.68) \| \| --- \| | | REF   \| 0.67 (0.42, 1.07) \| \| --- \| | REF  1.32 (0.89, 1.97) |
| ^a^Model adjusted for recruitment site, maternal age, race/ethnicity, household annual income, education, pre-pregnancy BMI, GA at sample collection, child adjusted age at CBCL administration, season, infant birth order. Note: OPE, Organophosphate Ester; T1, Tertile 1; T2, Tertile 2; T3, Tertile 3; CBCL, Child Behavior Checklist; DPHP, Diphenyl phosphate; DNBP+DIBP, Sum of Di-n-butyl phosphate and Di-isobutyl phosphate; BDCIPP, Bis(1,3-dichloro-2-propyl) phosphate; BCEP, Bis(2-chloroethyl) phosphate; BBOEP, Bis(butoxethyl) phosphate; BCIPP, Bis(1-chloro-2-propyl) phosphate; BMPP, Bis(2-methylphenyl) phosphate; BEHP, Bis(2-ethylhexyl) phosphate; DPRP, Dipropyl phosphate; GA, gestational age; BMI, Body Mass Index.  All $\beta's$ have been exponentiated for interpretation. | | | | | | | |

| Supplemental Table 5: Individual Associations Between Third Trimester Urinary OPE Metabolites (ng/mL) and CBCL Composite T-Scores (N=204) | | | | | | | | | | | |
| --- | --- | --- | --- | --- | --- | --- | --- | --- | --- | --- | --- |
|  |  | Internalizing | | | | Externalizing | | | Total Problems | |  |
|  | Unadjusted  $\beta$ (95% CI) | | Adjusted^a^  $\beta$ (95% CI) | Unadjusted  $\beta$ (95% CI) | | | Adjusted^a^  $\beta$ (95% CI) | Unadjusted  $\beta$ (95% CI) | | Adjusted^a^  $\beta$ (95% CI) | |
| DPHP  T1 (<0.55)  T2 (0.55-1.15)  T3 (≥1.15) | REF  0.99 (0.91, 1.07)  1.03 (0.94, 1.12) | | REF  1.00 (0.92, 1.09)  1.03 (0.94, 1.12) | REF  1.00 (0.93, 1.09)  1.04 (0.97, 1.13) | | | REF  1.00 (0.93, 1.09)  1.03 (0.95, 1.11) | REF  0.99 (0.92, 1.08)  1.04 (0.96, 1.13) | | REF  1.00 (0.92, 1.09)  1.03 (0.94, 1.12) | |
| DNBP+DIBP  T1 (<0.14)  T2 (0.14-0.21)  T3 (≥0.21) | REF  0.99 (0.91, 1.07)  1.00 (0.92, 1.09) | | REF  0.99 (0.91, 1.08)  1.01 (0.93, 1.10) | REF  1.03 (0.95, 1.11)  1.03 (0.96, 1.12) | | | REF  1.02 (0.94, 1.11)  1.03 (0.96, 1.12) | REF  1.01 (0.93, 1.10)  1.01 (0.93, 1.09) | | REF  1.01 (0.93, 1.09)  1.01 (0.93, 1.10) | |
| BDCIPP  T1 (<0.85)  T2 (0.85-1.83)  T3 (≥1.83) | REF  1.01 (0.92, 1.09)  0.98 (0.90, 1.07) | | REF  1.02 (0.93, 1.11)  1.01 (0.92, 1.11) | REF  1.02 (0.94, 1.10)  1.03 (0.95, 1.11) | | | REF  1.01 (0.93, 1.10)  1.03 (0.95, 1.13) | REF  1.01 (0.93, 1.10)  1.02 (0.94, 1.10) | | REF  1.01 (0.92, 1.10)  1.03 (0.94, 1.12) | |
| BCEP  T1 (Non-detect)  T2 (0.04-0.97)  T3 (≥0.97) | REF  1.02 (0.94, 1.11)  1.01 (0.93, 1.10) | | REF  1.03 (0.95, 1.12)  1.03 (0.94, 1.13) | REF  0.99 (0.92, 1.07)  0.99 (0.91, 1.07) | | | REF  1.01 (0.93, 1.09)  1.01 (0.93, 1.09) | REF  1.00 (0.92, 1.09)  1.00 (0.92, 1.08) | | REF  1.01 (0.93, 1.10)  1.01 (0.93, 1.10) | |
| BBOEP  T1 (Non-detect)  T2 (0.01-0.06)  T3 (≥0.06) | REF  1.07 (0.99, 1.17)  1.01 (0.93, 1.09) | | REF  1.07 (0.98, 1.16)  0.99 (0.91, 1.08) | REF  1.09 (1.01, 1.18)*  1.01 (0.94, 1.09) | | | REF  1.08 (1.00, 1.17)*  0.99 (0.92, 1.08) | REF  1.10 (1.01, 1.19)*  1.01 (0.93, 1.10) | | REF  1.09 (1.00, 1.18)*  1.00 (0.91, 1.08) | |
| BCIPP  T1 (Non-detect)  T2 (0.03- 0.66)  T3 (≥0.66) | REF  0.96 (0.88, 1.04)  1.04 (0.96, 1.13) | | REF  0.95 (0.87, 1.03)  1.05 (0.96, 1.14) | REF  0.99 (0.91, 1.07)  1.05 (0.97, 1.14) | | | REF  0.98 (0.91, 1.06)  1.04 (0.96, 1.13) | REF  0.97 (0.90, 1.06)  1.04 (0.96, 1.13) | | REF  0.97 (0.89, 1.06)  1.04 (0.96, 1.13) | |
| BMPP  Non-detect  Detect | REF  1.08 (1.01, 1.16)* | | REF  1.08 (1.01, 1.16)* | | REF  1.06 (0.99, 1.13) | | REF  1.07 (1.00, 1.14) | REF  1.08 (1.01, 1.15)* | | REF  1.08 (1.01, 1.16)* | |
| BEHP  Non-detect  Detect | REF  1.04 (0.96, 1.13) | | REF  1.03 (0.95, 1.12) | | REF  1.02 (0.95, 1.10) | | REF  1.01 (0.94, 1.08) | REF  1.03 (0.96, 1.12) | | REF  1.02 (0.94, 1.10) | |
| DPRP  Non-detect  Detect | REF  0.99 (0.92, 1.07) | | REF  1.00 (0.92, 1.08) | | REF  1.03 (0.96, 1.11) | | REF  1.03 (0.96, 1.11) | REF  1.02 (0.94, 1.10) | | REF  1.02 (0.95, 1.10) | |
| ^a^Model adjusted for recruitment site, maternal age, race/ethnicity, household annual income, education, pre-pregnancy BMI, GA at sample collection, child adjusted age at CBCL administration, season, infant birth order, child sex.  Note: OPE, Organophosphate Ester; T1, Tertile 1; T2, Tertile 2; T3, Tertile 3; CBCL, Child Behavior Checklist; DPHP, Diphenyl phosphate; DNBP+DIBP, Sum of Di-n-butyl phosphate and Di-isobutyl phosphate; BDCIPP, Bis(1,3-dichloro-2-propyl) phosphate; BCEP, Bis(2-chloroethyl) phosphate; BBOEP, Bis(butoxethyl) phosphate; BCIPP, Bis(1-chloro-2-propyl) phosphate; BMPP, Bis(2-methylphenyl) phosphate; BEHP, Bis(2-ethylhexyl) phosphate; DPRP, Dipropyl phosphate; GA, gestational age; BMI, Body Mass Index.  All $\beta's$ have been exponentiated to facilitate interpretation. | | | | | | | | | | | |

| Supplemental Table 6: Individual Associations Between Third Trimester Urinary OPE Metabolites (ng/mL) and CBCL Raw Composite Scores Among Participants Who Reported No In-Utero Smoking (N= 199) | | | |
| --- | --- | --- | --- |
|  | Internalizing  $\beta$ (95% CI) | Externalizing  $\beta$ (95% CI) | Total Problems  $\beta$ (95% CI) |
| DPHP  T1 (<0.55)  T2 (0.55-1.15)  T3 (≥1.15) | REF  0.88 (0.55, 1.41)  1.01 (0.62, 1.62) | REF  0.84 (0.58, 1.23)  0.98 (0.66, 1.44) | REF  0.86 (0.62, 1.18)  1.01 (0.72, 1.41) |
| DNBP+DIBP  T1 (<0.14)  T2 (0.14-0.21)  T3 (≥0.21) | REF  1.05 (0.65, 1.70)  1.17 (0.73, 1.88) | REF  1.01 (0.68, 1.48)  1.11 (0.76, 1.64) | REF  0.95 (0.68, 1.33)  1.06 (0.76, 1.48) |
| BDCIPP  T1 (<0.85)  T2 (0.85-1.83)  T3 (≥1.83) | REF  1.00 (0.61, 1.65)  1.00 (0.59, 1.67) | REF  1.01 (0.67, 1.51)  1.24 (0.82, 1.89) | REF  1.03 (0.73, 1.46)  1.18 (0.82, 1.68) |
| BCEP  T1 (Non-detect)  T2 (0.04-0.97)  T3 (≥0.97) | REF  1.18 (0.74, 1.90)  1.15 (0.70, 1.89) | REF  1.13 (0.77, 1.66)  1.04 (0.70, 1.55) | REF  1.03 (0.74, 1.43)  1.04 (0.74, 1.46) |
| BBOEP  T1 (Non-detect)  T2 (0.01-0.06)  T3 (≥0.06) | REF  1.22 (0.76, 1.94)  0.74 (0.45, 1.20) | REF  1.37 (0.94, 1.99)  0.79 (0.54, 1.17) | REF  1.22 (0.88, 1.68)  0.78 (0.56, 1.09) |
| BCIPP  T1 (Non-detect)  T2 (0.03- 0.66)  T3 (≥0.66) | REF  0.73 (0.45, 1.17)  1.49 (0.93, 2.39) | REF  0.90 (0.61, 1.32)  1.35 (0.92, 1.98) | REF  0.86 (0.62, 1.19)  1.22 (0.88, 1.70) |
| BMPP  Non-detect  Detect | REF  1.49 (1.00, 2.21)* | REF  1.46 (1.06, 2.01)* | REF  1.38 (2.05, 1.81)* |
| BEHP  Non-detect  Detect | REF  1.14 (0.72, 1.79) | REF  1.01 (0.70, 1.46) | REF  1.10 (0.81, 1.51) |
| DPRP  Non-detect  Detect | REF  0.87 (0.56, 1.35) | REF  1.12 (0.79, 1.60) | REF  0.98 (0.73, 1.33) |
| All models adjusted for recruitment site, maternal age, race/ethnicity, household annual income, education, pre-pregnancy BMI, GA at sample collection, child adjusted age at CBCL administration, season, infant birth order, child sex. Note: OPE, Organophosphate Ester; T1, Tertile 1; T2, Tertile 2; T3, Tertile 3; CBCL, Child Behavior Checklist; DPHP, Diphenyl phosphate; DNBP+DIBP, Sum of Di-n-butyl phosphate and Di-isobutyl phosphate; BDCIPP, Bis(1,3-dichloro-2-propyl) phosphate; BCEP, Bis(2-chloroethyl) phosphate; BBOEP, Bis(butoxethyl) phosphate; BCIPP, Bis(1-chloro-2-propyl) phosphate; BMPP, Bis(2-methylphenyl) phosphate; BEHP, Bis(2-ethylhexyl) phosphate; DPRP, Dipropyl phosphate; GA, gestational age; BMI, Body Mass Index.  All $\beta's$ have been exponentiated to facilitate interpretation. | | | |

| Supplemental Table 7: Median Concentrations (ng/mL) of Urinary OPE Metabolites Across Published Studies | | | | | | | | | | | | | |
| --- | --- | --- | --- | --- | --- | --- | --- | --- | --- | --- | --- | --- | --- |
| Country (City/State) | Cohort (N) | BDCIPP | DPHP | BCIPP | DNBP  +  DIBP | BCEP | BBOEP | BMPP | BEHP | DPRP | Adjustment | Population  (GA at Collection/Age) | Reference |
| **US**  **(Los Angeles /CA)** | **MADRES (N=204)** | **1.26** | **0.83** | **0.04** | **0.17** | **0.47** | **0.04** | **0.01** | **ND** | **ND** | **SG adjusted** | **Birth cohort (31.4 (SD: 1.8) weeks)** | **Our Study** |
| Norway (Oslo) | MoBa (N=555 subcohort) | <0.17 | 0.45 | - | - | - | 0.07 | - | - | - | SG adjusted | Birth cohort 17 weeks | Choi et al., 2021 |
| US (North Carolina) | Pregnancy, Infection, Nutrition (PIN) (N=199) | 2.01 | 1.38 | _ | _ | _ | _ | _ | _ | _ | SG adjusted | Birth cohort (27 weeks (range 24-29)) | Doherty et al., 2019 |
| US (Salinas/CA) | Center for the Health Assessment of Mothers and Children of Salinas (CHAMACOS) (N=310) | 0.41 | 0.93 | _ | _ | _ | _ | _ | _ | _ | SG adjustment | Birth cohort (26.0 (2.4) weeks) | Castorina et al., 2017 |
| US (Baltimore, MD) | ORigins of Child Health And Resilience in Development (ORCHARD) (N=90) | 0.51 | 1.12 | ND | _ | _ | _ | _ | _ | _ | SG adjustment | Birth cohort (30.9 (SD: 2.5) weeks) | Kuiper et al., 2020 |
| US (Rhode Island) | Women and Infants Hospital of Rhode Island (N=56) | 1.18 | 0.93 | _ | _ | 0.31 | _ | _ | _ | _ | SG adjustment | Birth cohort (1^st^-3^rd^ trimester) | Crawford et al., 2020 |
| China (Wuhan) | Wuhan Maternal and Child Healthcare Hospital (N=213) | 0.10 | 0.24 | _ | _ | _ | 0.14 | _ | _ | _ | SG adjusted | Birth cohort (1^st^-3^rd^ trimester) | Luo et al., 2021 |
| US (Boston/Massachusetts) | LIFECODES (N= 90) | 0.67 | 0.74 | _ | _ | _ | _ | _ | _ | _ | SG adjusted | Birth cohort (1^st^-3^rd^ trimester) | Bommarito, et al., 2021 |
| Note: GA, Gestational Age; OPE, Organophosphate Ester; DPHP, Diphenyl phosphate; DNBP+DIBP, Sum of Dibutyl phosphate and Di-isobutyl phosphate; BDCIPP, Bis(1,3-dichloro-2-propyl) phosphate; BCEP, Bis(2- chloroethyl) phosphate; BBOEP, Bis(butoxethyl) phosphate; BCIPP, Bis(1-chloro-2-propyl) phosphate; BMPP, Bis(2-methylphenyl) phosphate; BEHP, Bis(2-ethylhexyl) phosphate; DPRP, Dipropyl phosphate. | | | | | | | | | | | | | |
